# Recombination in Enteroviruses Is a Biphasic Replicative Process Involving the Generation of Greater-than Genome Length ‘Imprecise’ Intermediates

**DOI:** 10.1371/journal.ppat.1004191

**Published:** 2014-06-12

**Authors:** Kym Lowry, Andrew Woodman, Jonathan Cook, David J. Evans

**Affiliations:** School of Life Sciences, University of Warwick, Coventry, United Kingdom; University of Colorado Health Sciences Center, United States of America

## Abstract

Recombination in enteroviruses provides an evolutionary mechanism for acquiring extensive regions of novel sequence, is suggested to have a role in genotype diversity and is known to have been key to the emergence of novel neuropathogenic variants of poliovirus. Despite the importance of this evolutionary mechanism, the recombination process remains relatively poorly understood. We investigated heterologous recombination using a novel reverse genetic approach that resulted in the isolation of intermediate chimeric intertypic polioviruses bearing genomes with extensive duplicated sequences at the recombination junction. Serial passage of viruses exhibiting such imprecise junctions yielded progeny with increased fitness which had lost the duplicated sequences. Mutations or inhibitors that changed polymerase fidelity or the coalescence of replication complexes markedly altered the yield of recombinants (but did not influence non-replicative recombination) indicating both that the process is replicative and that it may be possible to enhance or reduce recombination-mediated viral evolution if required. We propose that extant recombinants result from a biphasic process in which an initial recombination event is followed by a process of resolution, deleting extraneous sequences and optimizing viral fitness. This process has implications for our wider understanding of ‘evolution by duplication’ in the positive-strand RNA viruses.

## Author Summary

The rapid evolution of most positive-sense RNA viruses enables them to escape immune surveillance and adapt to new hosts. Genetic variation arises due to their error-prone RNA polymerases and by recombination of viral genomes in co-infected cells. We have developed a novel approach to analyse the poorly understood mechanism of recombination using a poliovirus model system. We characterised the initial viable recombinants and demonstrate the majority are longer than genome length due to an imprecise crossover event that duplicates part of the genome. These viruses are unfit, but rapidly lose the duplicated material and regain full fitness upon serial passage, a process we term resolution. We show this is a replicative recombination process by modifying the fidelity of the viral polymerase, or replication complex coalescence, using methods that have no influence on a previously reported, less efficient, non-replicative recombination mechanism. We conclude that recombination is a biphasic process involving separate generation and resolution events. These new insights into an important evolutionary mechanism have implications for our understanding of virus evolution through partial genome duplication, they suggest ways in which recombination might be modified and provides an approach that may be exploited to analyse recombination in other RNA viruses.

## Introduction

The high levels of genetic variation observed in many RNA viruses has important consequences for viral pathogenesis, host tropism and evolution. Two predominant mechanisms contribute to the generation of genetic diversity of the genome; misincorporation of nucleotides and recombination/reassortment. The absence of proofreading by the majority of viral RNA-dependent RNA-polymerases (RdRp) results in error frequencies of 10^−3^ to 10^−5^ per nucleotide polymerized [1], so generating a population of related genomes, the quasispecies [2]. In contrast to the incremental changes (‘drift’) that accumulate from this misincorporation, much more extensive variation can result from exchange or acquisition of large regions of the virus genome through the processes of recombination or, in terms of genetic consequences for the segmented RNA viruses, the analogous process of reassortment.

**Figure 1:**
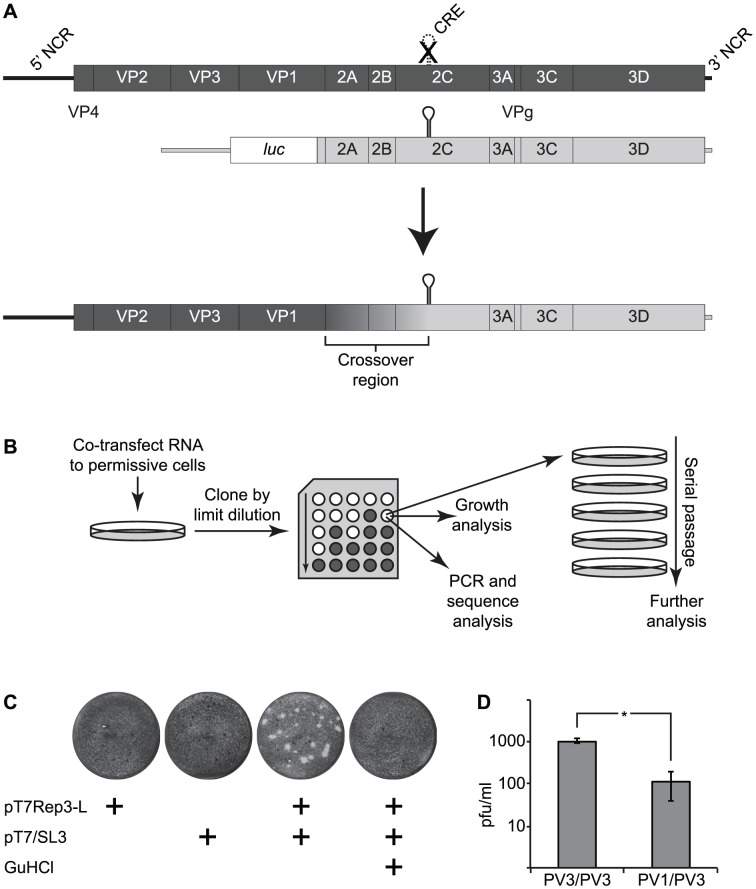
The CRE-REP assay to analyse recombination in enteroviruses. A) Schematic diagram of enterovirus recombination partners and products. The upper panel shows a diagram of a genome (dark shading) bearing a defective CRE indicated as a broken line with a superimposed X in the 2C-coding region and a luciferase-encoding replicon (light shading). Following co-transfection into permissive cells (indicated by an arrow), a replication competent recombinant genome may be recovered of the generic structure shown, consisting of the 5′ part of the genome derived from the CRE-defective parent (the recipient) and the 3′ part of the genome from the luciferase-encoding replicon (the donor). The graduated shading between the 3′ end of the VP1-coding region and the 2C CRE indicates the area within which recombination must occur to produce a functional genome. B) Schematic diagram of the recombinant production strategy. RNA generated *in vitro* was co-transfected into cells permissive for virus replication but not susceptible to infection. After 24–48 hours, supernatant virus was recovered, cloned by limit dilution and subjected to further analysis. C) Xylene cyanol-stained HeLa cell monolayers infected with supernatant from murine L929 cells transfected with RNA derived from the plasmids indicated. Guanidine hydrochloride (GuHCl; 4 mM) added where indicated to HeLa cells. D) Comparison of inter- and intra-serotypic recombination following independent co-transfection of L929 murine fibroblasts with RNA generated from poliovirus type 3 (PV3/PV3) or poliovirus type 1 and type 3 (PV1/PV3) cDNAs. The error bars indicate the standard deviation of three independent assays with significance determine by chi square test (*p* = 0.01).

The enteroviruses are a well characterised genus within the family *Picornaviridae*. The human enteroviruses (HEV) are particularly well studied as they contain important pathogens responsible for acute flaccid paralysis (including poliomyelitis), myocarditis, encephalitis and a variety of other ailments [3] and have recently been extended to include the three species of human rhinoviruses [4]. The nonsegmented single-stranded positive-sense enterovirus RNA genome is ∼7.5 kb in length, is polyadenylated at the 3′ end and encodes a single polyprotein from an open reading frame flanked by an extensive 5′ and shorter 3′ non-coding region (NCR). The polyprotein is co- and post-translationally proteolytically processed to yield (in order of translation) the viral structural (VP4, 2, 3 and 1; occupying the P1 region, Figure 1) and non-structural proteins (2A^pro^, 2B, 2C, 3A, VPg, 3C^pro^ and 3D^pol^; comprising the P2 and P3 regions of the genome). The latter control the cellular environment, establish the membrane-bound replication complex, replicate the genome via formation of a negative-stranded intermediate and process the polyprotein.

The HEV are divided into four phylogenetically distinct species (designated A–D; [4]) the members of which were initially differentiated serologically, and are now usually defined by sequence analysis of VP1 [5]–[8]. Extensive sequencing studies have unequivocally demonstrated the time-related accumulation of sequence variation – and consequent antigenic diversification – within the capsid-coding region [9]–[14] and frequent recombination within the regions encoding the non-structural proteins [13], [15]–[33]. Additional recombination events have been characterised between the 5′ NCR and capsid coding region [34], [35]. The HEV genome can therefore be considered as modular, with recombination between the functional entities that drive translation and replication (5′ NCR), that encode the structural proteins (the P1 region) and that encode the non-structural proteins [21], [28]. Recombination within the P1 region has rarely been observed, and only then between very closely related viruses or involving the extreme termini of VP1, presumably due to restrictions imposed on assembly of the icosahedral particle [36]. In contrast, in addition to the extensive documentation of recombination within the non-structural protein coding region, different serotypes exhibit distinct temporal and geographic kinetics in the appearance of the predominant recombinant forms (RF) [23]–[25] although the selection of emergent RFs, whether due to replicative or immunological advantage, has yet to be determined [25].

The majority of characterised HEV recombinants circulating are intraspecies, though interspecies recombination may have occurred in ancestral enteroviruses [37]–[40] and 5′ NCR exchanges constructed in the laboratory can generate viable viruses [41], [42]. The restrictions preventing viable interspecies recombinants between structural and non-structural coding regions are poorly understood. In addition to a requirement to access the same cell type and co-occupy the same replication complex [43], these presumably include the necessity for compatibility in all *cis*-acting replication functions [44]. For example, recent evidence indicates that particle morphogenesis requires an interaction of the 2C protein and capsid protein VP3 [45], [46].

Despite the importance of recombination as an evolutionary process in enteroviruses, the molecular mechanism involved has received relatively little attention. The favoured replicative mechanism in enteroviruses (and other RNA viruses) involves template-switching of the viral RdRp during negative strand RNA synthesis [47]–[49]. An alternative process, involving the replication-independent joining of RNA molecules has been described by Agol and colleagues [50], [51] which may be mediated by cellular RNA ligases as postulated for analogous studies in pestiviruses and hepatitis C virus [52], [53].

Studies of the viable recombinant progeny from cells co-infected with enteroviruses are confounded by the relatively low frequency with which recombinants are generated and the high levels of parental viruses generally produced from such infections. Together, this necessitates the use of a selection strategy to preferentially isolate recombinants from a mixed population, which likely imposes an additional selection for increased fitness within the recombinant population. To overcome these limitations we have developed an *in vitro* reverse genetic system that enables the recovery of recombinants alone from dually transfected cells in culture. We have used this to characterise the recombination junctions and subsequently investigate the replication phenotype and stability of the recovered viruses. Our studies suggest that replicative (*i.e*. polymerase-dependent) recombination is a biphasic process in which an initial chimeric genome is generated that contains a duplication of up to several hundred nucleotides (nt.) of the virus genome. Subsequent replication of these genomes results in a process of resolution in which variants are selected that have undergone an internal deletion to remove the duplicated sequences, resulting in genomes of the expected (*i.e*. genomic) length. These studies provide important insights into the molecular mechanism of recombination in positive strand RNA viruses and additionally suggest a means by which ‘evolution by duplication’ may occur. The novel experimental strategy we described provides the basis for further studies of the viral and cellular processes that control this important evolutionary process.

## Results

### A system for generating recombinant enteroviruses

To facilitate the analysis of recombination junctions and early recombination events in enteroviruses we wanted an approach that allowed the production of recombinants in the absence of either replication-competent helper viruses or low frequency revertants of parental genomes bearing genetic lesions such as resistance to guanidine hydrochloride [45], [54]. Since recombination is proposed to occur by a ‘copy-choice’ process involving polymerase template switching during negative strand synthesis [47] we reasoned that one of the parental genomes could be defective in synthesis of positive-strands, a phenotype associated with destabilization of the *cis*-acting replication element (CRE) located within the 2C coding region of enteroviruses [55]. We have previously demonstrated that a poliovirus genome bearing eight synonymous substitutions within this CRE does not revert, even after extensive blind passage [56], [57]. For the second parental genome we used a replication-competent sub-genomic replicon, in which the capsid-coding region was replaced with a luciferase reporter gene. The rationale was that co-transfection of RNA generated *in vitro* from these two cDNAs should yield a complete, replication competent genome – bearing both the capsid-coding region and a functional CRE – if a recombination cross-over occurred between the end of the capsid coding region and the CRE (Figure 1a). The reciprocal recombinant – if generated – bearing a luciferase reporter and non-functional CRE would not replicate and neither parental genome was capable of generating progeny virus. To facilitate the recovery of potential recombinants that had not undergone successive rounds of release, re-infection and replication, we used rodent cells – permissive for virus replication but lacking a suitable receptor and so not susceptible to infection – for transfection. This approach facilitated the capture of early recombinant virus progeny whilst minimizing their loss due to continued propagation. The generic recombination system and subsequent isolation strategy is illustrated in Figure 1b. For convenience, and because it refers to the parental genomes used in the assay, we designate this as a CRE-REP recombination assay.

We initially investigated the ability to generate intra-serotypic recombinants using parental genomes derived from poliovirus type 3 as we reasoned that – if sequence identity or protein-protein compatibility influenced the efficiency with which viable recombinants could be recovered – this would impose the minimum constraints on the process. L929 murine fibroblasts were transfected – individually or together – with RNA generated *in vitro* from pT7Rep3-L and pT7/SL3. Cell-free supernatant was harvested 24–48 hours post-transfection and the presence of infectious virus detected by plaque assay in HeLa cells. Individually, neither parental genome yielded virus capable of forming plaques on the HeLa monolayers. In contrast, undiluted supernatant from co-transfected L929 cells yielded ∼1.1×10^3^ pfu/ml of virus (Figure 1cd), the identity of which was confirmed by PCR analysis and subsequent sequencing. L929 cells yielded no recombinants when the growth media was supplemented with 4 mM guanidine hydrochloride, a known inhibitor of poliovirus negative strand initiation [58].

We sequenced several biologically cloned intra-serotypic recombinants (see below). However, the inevitable sequence identity of the parental viral genomes confounded the identification of recombination junctions in the majority of recovered viruses. Therefore, having demonstrated the recovery of viable progeny after co-transfection of a sub-genomic replicon and CRE mutant we repeated the experiment replacing the poliovirus type 3-derived luciferase-encoding sub-genomic replicon with an equivalent replicon derived from poliovirus type 1 (plasmid pRLucWT; 21% divergent within the potential recombination region shown in Figure 1). In this instance co-transfection of both parental plasmids routinely generated 50–200 pfu/ml (mean of 115 pfu/ml from three independent replicates) from transfected murine L929 cells following transfer of undiluted supernatant to HeLa cell monolayers (Figure 1d). In repeated independent co-transfections of mouse or hamster (L929 or BHK respectively; not shown) monolayers, homologous (PV3/PV3) parental genomes yielded ∼10-fold more progeny than heterologous (PV1/PV3) parental genomes (P<0.05 Mann Whitney U test; Figure 1d). Inter-serotypic poliovirus recombinants were biologically cloned by limit dilution and analysed further.

### Sequence analysis of poliovirus recombinants

**Figure 2:**
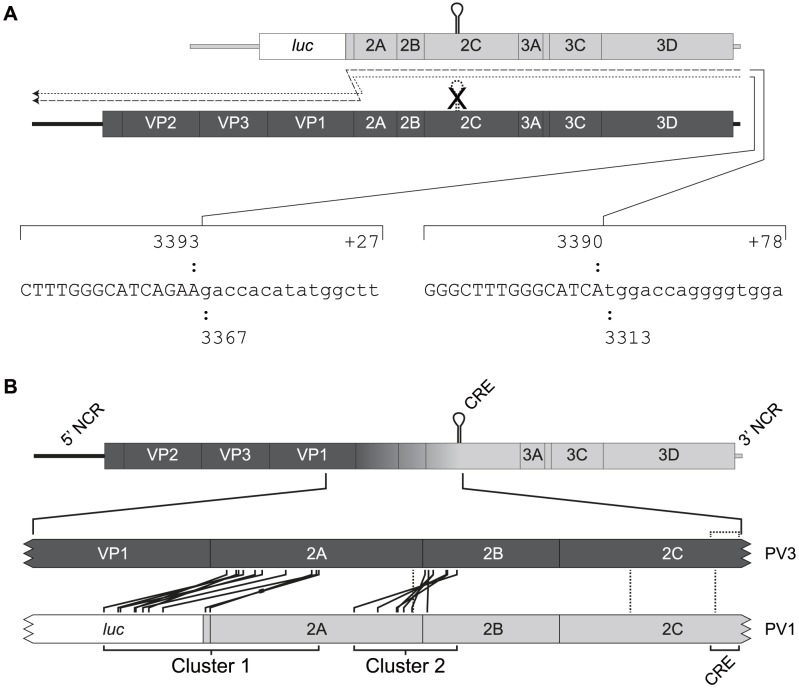
Recombination generates imprecise junctions with genome duplications. A) Imprecise recombinants recovered following co-transfection of murine L929 cells with RNA generated *in vitro* from pT7Rep3-L (upper genome) and pT7/SL3 (lower genome). The junction sequence of two imprecise recombinants, indicated with dashed lines, are shown. Upper and lower case characters are used to distinguish between those derived from the CRE-defective genome (pT7/SL3) and sub-genomic replicon (pT7Rep3-L) respectively, with the extent of the sequence numbered according to the reference genomes (see Materials and Methods). For each imprecise recombinant the number prefixed with a plus sign indicates the additional nucleotides incorporated at the junction. B) Schematic diagram showing the location of recombinant junctions characterised following co-transfection of murine L929 cells with CRE-defective type 3 poliovirus genome (pT7/SL3; dark shading) and poliovirus type 1 sub-genomic replicon RNA (pRLucWT; light shading). The graduated shading in the upper genome indicates the region within which recombination must have occurred to generate viable progeny virus. The expanded portion of the genomes (below) indicate the position of the junctions in the polymerase donor (pRLucWT) and recipient (pT7/SL3) genomes. Solid lines indicate the groups of imprecise junctions – defined by sequence – which are grouped into two clusters spanning the P1/P2 and 2A/2B boundaries. The thickened line in one of the cluster 1 imprecise recombinants indicates the genome bearing additional sequence derived from the 5′ NCR (#25A see Figure 3). Precise recombinants are indicated with vertical dotted lines within the 2A and 2C coding regions.

Fifteen intra-serotype recombinants were analysed after co-transfection of L929 monolayers with RNA generated *in vitro* from pT7Rep3-L and pT7/SL3. Recovered virus was reverse transcribed using an oligo-dT primer and the region within which a recombination event was targeted to occur was amplified using oligonucleotides PV3-2995F and PV3-5191R (Supplementary Table S1) and subsequently sequenced. The majority (n = 13) of the recovered genomes were indistinguishable from the parental type 3 poliovirus genome. However, to our surprise two genomes included a duplication, of either 27 nt. (PV3^3393^/PV3^3367^; see Materials and Methods for nomenclature used to define recombinants and recombination junctions) or 78 nt. (PV3^3390^/PV3^3313^) within the region encoding the 2A protease (Figure 2a). We designated these types of recombinants as *imprecise* reflecting the nature of the junction between the donor (assumed to be the 3′ partner encoding the viral polymerase) and recipient parental genomes. Since further analysis of such imprecise recombinants was limited by the sequence identity of the parental viruses we went on to analyse the products from a PV1/PV3 inter-serotypic co-transfection.

A total of 146 viable viruses were biologically cloned by limit dilution of cell-free supernatant after three independent co-transfections of L929 cells with equimolar ratios of RNA generated *in vitro* from pRLucWT and pT7/SL3. Following reverse transcription, the oligonucleotides PV3-2995F and PV1-5200R (Supplementary Table S1) were used to amplify the intervening region. Initial analysis by gel electrophoresis indicated that a significant proportion of the products were larger that the expected size of 2205 nt. (data not shown). All amplified products were therefore sequenced. Of these, 10 were discarded as the sequence was ambiguous indicating they did not contain a clonal virus population. The remaining 136 viruses all consisted of recombinants in which the 5′ component was derived from the PV3 cDNA pT7/SL3 and the 3′ component was derived from the PV1 sub-genomic replicon, pRLucWT. These were stratified – on the basis of the unique sequence that defined the junction – into 20 distinct groups (illustrated schematically in Figure 2b; Figure S1). Of these, three groups (#4E, #35C and #44B) contained a recombination junction in which no additional sequences were present, located in either the region encoding the C-terminus of 2A^pro^ (#35C) or the 2C protein (#35C and #44B; in the latter group the junction was defined by the mutation engineered into the SL3 parental virus [56]). We designate these as *precise* junctions *i.e*. in a sequence alignment of the two parental genomes, the locations of the 3′ nucleotide of the recipient genome and the 5′ nucleotide of the donor are adjacent. The remaining 17 distinct recombination groups contained junctions in which additional sequences of 3 to 321 nt. were present (*i.e. imprecise* junctions). All imprecise junctions maintained the open reading frame. In eight groups the 5′ nucleotide of the donor was located within the luciferase encoding region of the PV1-derived sub-genomic replicon. These groups, together with groups #32A (in which the PV1 sequence started within the linker between the reporter gene and the 2A^pro^-coding region; Figure 2b and Figure S1) and #25A (see below), contained an average 254 additional nucleotides and formed a distinct cluster (Cluster 1) in which the imprecise junction spanned the region encoding the VP1/2A^pro^ cleavage site (Figure 2b). A second cluster (Cluster 2), containing six groups with an average 118 additional nucleotides had imprecise recombination junctions spanning the region encoding the 2A^pro^/2B cleavage site. We included group #9C, which contains a 3 nt. duplication within the region encoding the extreme amino-terminus of the 2B protein, in Cluster 2. In six of the recombination groups it was not possible to unambiguously define the recombination junction due to local sequence identity between the aligned parental genomes at the junction (#11A, #34D, #53A, #67B, #E1 and #4E; Figure S1). Only one group (#25A) contained virus-derived sequences that were not an effective duplication of adjacent sequences at an imprecise junction. This group additionally contained 16 nts. derived from the 5′ NCR (nts. 447–462) of a total of 243 nts. additional sequence present within the crossover region (Figure S1). Since this region of the 5′ NCR was identical in the parental genomes it was not possible to determine which it had been derived from.

### Growth characteristics and serial passage of recombinant viruses

Of the 136 heterotypic recombinants sequenced, the majority – both in terms of individual sequences (95/136) or distinct groups (17/20) – contained additional sequences within the recombination region (Figures 2b and S1). Since recombinant polioviruses previously reported do not contain additional sequences we reasoned that these imprecise recombinants might have a growth disadvantage. We therefore investigated the growth characteristics and stability of the virus genome upon serial passage of selected recombinants.

**Figure 3:**
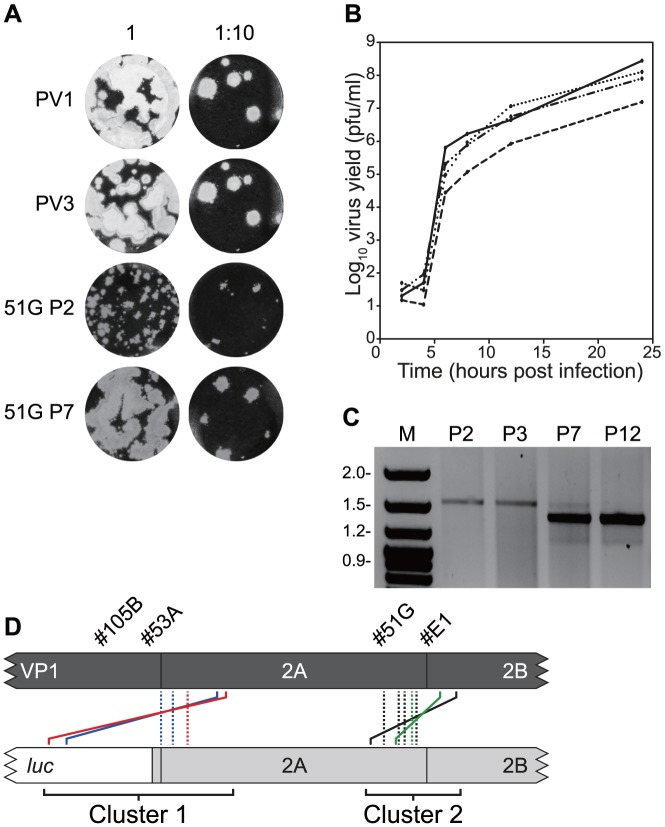
Serial passage of recombinants with imprecise junctions generate viruses with increased fitness bearing precise junctions. A) Plaque phenotypes in HeLa cell monolayers of parental (PV1, PV3) and recombinant viruses (#51G) following two (P2) or seven (P7) passages of the latter in HeLa cells. B) Single step growth analysis over 24 hours of parental viruses (PV1, solid line; PV3, dotted line) and #51G imprecise recombinant virus before (dashed line) and after (mixed dotted and dashed line) seven serial passages is HeLa cells. Virus titre (in pfu/ml) shown is the average of three independent assays. C) RT-PCR analysis of the junction region in recombinant #51G during serial passage in HeLa cells. Viral RNA was reverse transcribed using an oligo-dT primer and cDNA amplified using primers PV3-2995F and GEN-4615R. The resulting products were analysed by horizontal agarose gel electrophoresis after staining with ethidium bromide. D) Schematic diagram of precise recombinants derived by serial passage of the imprecise recombinants indicated. Solid lines (#105B  =  blue, #53A  =  red, #51G  =  green, #E1  =  black) indicate the original imprecise recombinant with colour-matched dashed lines indicating the position of the resulting precise junctions.

A recombinant from group #51G (PV3^3874^/PV1^2785^, cluster 2; Figure S1) was passaged a dozen times in HeLa cells. The plaque phenotype and single step growth characteristics of the second (P2) and seventh (P7) passage were compared with the parental poliovirus type 1 and type 3 (Figure 3ab). The initial small plaque phenotype (P2) increased by P7 to one close to that of either poliovirus types 1 or 3. In a single step growth analysis the P7 virus was indistinguishable from poliovirus type 1 or type 3, whereas the P2 virus had a lower yield at all time points tested, with a titre at 24 hours ∼1 log_10_ reduced. This suggested that serial passage of #51G resulted in changes that favoured the selection of viruses with faster replication kinetics and a large plaque phenotype. RNA therefore was purified at P2, P3, P7 and P12, reverse transcribed and the region spanning the recombination junction amplified by PCR using oligonucleotides PV3-2995F and GEN-4615R. In early passages (P2, P3) a single product was amplified of ∼1.7 kb but was largely replaced by P7 (and totally by P12) with a PCR product of a reduced size (Figure 3c). The P7 product was cloned and eight resulting cDNAs selected at random were sequenced.

Four distinct junction sequences between poliovirus types 1 and 3 were identified (Figure 3d; #51G-a to –d in Figure S2). In each case the product contained a precise junction in which there were no additional duplicated sequences from either parental genome. The four distinct junctions all mapped within the limits of overlap in the original recombinant PV3^3874^/PV1^2785^ and to the region of the genome encoding the C-terminus of 2A^pro^ (Figure 3d). In each case it was not possible to unambiguously identify the junction due to identity of 2–4 nucleotides at the crossover region.

Three further recombinants, one more from cluster 2 (#E1; PV3^3849^/PV1^2830^) and two from cluster 1 (#53A, PV3^3485^/PV1^2256^ and #105B, PV3^3471^/PV1^2287^) were similarly analysed. In each instance, serial passage (6 to 12 times) in HeLa cells resulted in the internal deletion of sequences present within the region duplicated in the imprecise junction, confirmed by sequence analysis of 8 randomly selected cDNAs (Figure 3d). All eight sequences recovered from recombinant #E1 were identical, as were those from #53A, whereas recombinant #105B yielded two distinct precise junctions (Figure S2). As before, in all cases the exact recombination junction could not be defined due to the conservation of 2–13 nucleotides at the junction (Figure S2). We interpreted these results as indicating that the recombination process in enteroviruses is biphasic – the initial generation of an imprecise recombinant followed by the selection of precise recombinants with enhanced fitness.

### Construction and selection of an imprecise recombinant

The process by which initial recombinant genomes were generated – cloning by limit dilution with minimal passage – meant there was a possibility that they were not truly clonal but instead may have contained undetectable levels of precise recombinants. Potentially these subsequently out-competed the imprecise recombinant, rather than being derived from it. To exclude this possibility we constructed a complete cDNA for the #105B recombinant (PV3^3471^/PV1^2287^; see Materials and Methods) and transfected L929 cells with RNA transcribed *in vitro*. Supernatant media from transfected L929 cells contained ∼8×10^4^ pfu/ml of virus as quantified by plaque assay in HeLa cells. This virus population was subjected to further serial passage and analysed by PCR as before. After four passages a product indistinguishable in size from that of the parental poliovirus genome remained visible together with one or more additional smaller PCR products, intermediate in size between the starting material and the parental poliovirus genome (data not shown). After three further passages the amplified PCR product was cloned and six clones were sequenced. Of these, four (#105B-3; Figure S2) exhibited an identical imprecise junction, PV3^3381^/PV1^2389^, between the PV3- and PV1 replicon-derived sequences. This differed from that present in the input genome (#105B, PV3^3471^/PV1^2287^; Figure S1) but retained 57 nt. derived from the region encoding the C-terminus of the luciferase reporter gene. The two remaining sequences exhibited different precise junctions (#105B-1, #105B-2; Figure S2). One of these (#105B-1, PV3^3378^/PV1^2443^; Figure S2), exhibited an identical precise recombination junction to that selected previously by serial passage of recombinant viruses cloned by limit dilution from a co-transfection of PV3 and pRLucWT (#105B-a; Figure S2). These results strongly support our contention that the imprecise recombinants isolated from L929 cells using limited passage and serial dilution are the likely progenitors of the precise recombinants subsequently selected by serial passage in HeLa cells. Furthermore, the identification of an imprecise recombinant with a duplicate sequence reduced in length from the input (249 nt. in #105B *cf*. 57 nt. in #105B-3, see Figure S2) suggests that the generation of precise recombinants may progress via intermediate imprecise recombinants.

### Are recombinants generated by a replicative or non-replicative process?

The CRE-REP assay we have developed (Figure 1) was based on the assumption that recombination occurs via a copy-choice mechanism during negative strand synthesis. However, formally the mechanism could have been replication-independent, as has been reported for a number of positive strand RNA viruses including enteroviruses, BVDV and HCV [50], [52], [53]. To clarify this we investigated the consequences of modifying the polymerase error rate, reasoning that a replicative process might be influenced by the characteristics of the polymerase. We therefore investigated the frequency of recombinant generation in the presence of ribavirin, an antiviral which induces a well-characterised increase in the poliovirus polymerase errors rate [59]. We also determined the influence of a G64S substitution in the polymerase, a mutation that confers resistance to ribavirin [60] as a consequence of increased polymerase fidelity [61]. We additionally investigated the influence of the drug nocodazole on recombination as previous studies have shown it inhibits the formation of mixed replication complexes which we presumed are required for replicative recombination [62]. In each instance we also investigated the influence of these various treatments or modifications on the generation of recombinants in a non-replicative assay.

**Figure 4:**
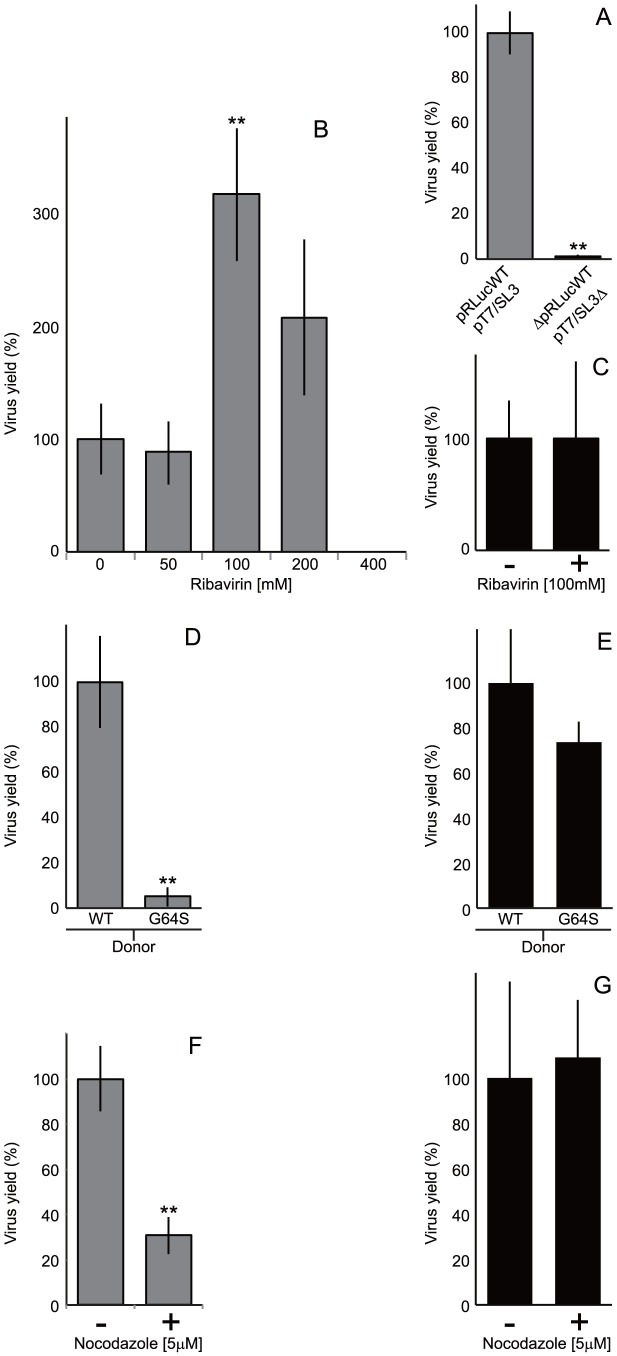
Comparison of the CRE-REP with non-replicative recombination assays and the influence of the viral polymerase on recombination. A) Recombinant virus yield in the CRE-REP and non-replicative recombination assays. Equimolar amounts of RNA generated *in vitro* from pT7/SL3 and pRLucWT or pT7/SL3Δ and ΔpRLucWT respectively – 0.5 µg total – were transfected into L929 cell monolayers. Viable progeny virus was harvested at 48 (CRE-REP) or 60 (non-replicative) hours post-transfection and quantified by plaque assay on HeLa cell monolayers. Virus yield is normalized to the yield in the CRE-REP assay, with error bars indicating standard deviation of three independent assays. B) Recombinant virus yield in the CRE-REP assay upon co-transfection of L929 cells in the presence of different concentrations of ribavirin. L929 cell monolayers were pre-treated with ribavirin at the concentrations indicated, transfected with RNA generated *in vitro* from pT7/SL3 and pRLucWT. After 48 hours in the presence of ribavirin, virus in the supernatant was quantified by plaque assay on HeLa cells. Results are presented (average of three independent assays with error bars indicating standard deviation) as the percentage with reference to transfected untreated monolayers, with statistical significance (** = *p*<0.001) determined by student t tests. C) The influence of 100 mM ribavirin on the yield of viable progeny virus from a non-replicative recombination assay. L929 cell monolayers pre-treated with 100 mM ribavirin were transfected with RNA generated *in vitro* from pT7/SL3Δ and ΔpRLucWT. After 60 hours, viable recombinant virus in the supernatant was quantified by plaque assay on HeLa cells. Results are normalized to the yield of progeny recombinant virus from the same truncated templates in the absence of ribavirin, with error bars indicating the standard deviation in three independent assays. D) Comparison of recombinant virus yield in the CRE-REP assay upon co-transfection of L929 cells with RNA generated *in vitro* from pT7/SL3 and RNA from either an unmodified (wildtype; WT) poliovirus type 1 sub-genomic replicon (pRLucWT) or a derivative bearing a high-fidelity G64S substitution in the viral 3D polymerase (pRLucWT_G64S_). Results are presented (average of three independent assays with error bars indicating standard deviation) as the percentage normalized to pRLucWT, with statistical significance (** = *p*<0.001) determined by student t tests. E) The influence of a G64S high fidelity polymerase mutation on the yield of viable progeny in a non-replicative recombination assay. As above, L929 cell monolayers were transfected with RNA generated *in vitro* from unmodified or G64S-bearing ΔpRLucWT templates and RNA from pT7/SL3Δ. Results presented are the percentage yield normalized to the unmodified truncated templates, with error bars indicating the standard deviation of three independent assays. F) The influence of nocodazole on recombinant yield in the CRE-REP assay. As before, RNA generated *in vitro* from pT7/SL3 and pRLucWT was transfected into L929 cells previously chilled and treated with 5 µM nocodazole (+; see Materials and Methods for details). Results are normalized to the yield from the same templates in the presence of DMSO carrier alone (-), with error bars indicating the standard deviation of three independent assays (** indicates p<0.005, student t tests). G) The influence of nocodazole on non-replicative recombination. RNA was generated *in vitro* from pT7/SL3Δ and ΔpRLucWT and transfected, as above, into nocodazole (+) or DMSO carrier-treated (–) L929 cell monolayers. The results show the percentage normalized yield to controls, with error bars indicating the standard deviation of three independent assays.

We initially compared the yield of intra-serotype recombinants in the CRE-REP assay we developed (Figure 1) with the yield from truncated – and therefore non-replicating – pT7/SL3Δ and ΔpRLucWT templates (see Materials and Methods for details). Using equimolar amounts of each RNA, and the same total amount of RNA for the CRE-REP or non-replicative transfection of L929 cells, progeny virus was quantified in the supernatant. Under these conditions, the yield of recombinants in the non-replicative assay was only 3.8% of the yield produced in the CRE-REP assay (Figure 4a). Ribavirin concentrations greater than 400 µM eliminated the generation of recombinant progeny following co-transfection of rodent cells with RNA derived from pT7/SL3 and pRLucWT, a result in agreement with previous data on the inhibitory concentration of ribavirin [59]. In contrast, concentrations of ribavirin of 200 µM and 100 µM increased the yield of progeny virus (over that produced in the absence of ribavirin) significantly, by ∼2 fold and ∼3 fold respectively (Figure 4b). However, 100 µM ribavirin did not influence the yield of progeny from a non-replicative assay (Figure 4c) using the truncated pT7/SL3Δ and ΔpRLucWT versions of the cDNA templates. Ribavirin concentrations over the range tested also had no influence on transfection efficiency of L929 cells (Figure S3).

In contrast to the enhancing effect of ribavirin use of donor and recipient genomes bearing a 3D^pol^ G64S high-fidelity substitution markedly reduced the yield of recombinants in the CRE-REP assay, by ∼20-fold (Figure 4d), despite the mutation having no discernible influence on poliovirus replication ([60]; Figure S4). In a parallel non-replicative recombination assay, using the truncated sub genomic replicon template bearing the same G64S polymerase mutation, there was no significant influence on the yield of progeny recombinants (Figure 4e).

Nocodazole treatment (5 µM) of transiently chilled cells prevents repolymerization of microtubules and the consequent coalescence of replication complexes, yet does not inhibit virus replication or yield ([62], [63]; Figure S5). In the CRE-REP assay, prior treatment of cells with nocodazole reduced the intra-serotypic yield of recombinants by ∼70% (Figure 4f). In contrast, similarly treated L929 cells transfected with RNA from the pT7/SL3Δ and ΔpRLucWT non-replicative versions of the same templates, generated similar levels of recombinant progeny to untreated cells (Figure 4g). Taken together, the influence of the polymerase characteristics and the requirement for replication complex mixing strongly suggests that the generation of recombinants in the CRE-REP assay involves a replicative process.

### Analysis of imprecise and precise recombination junctions

**Figure 5:**
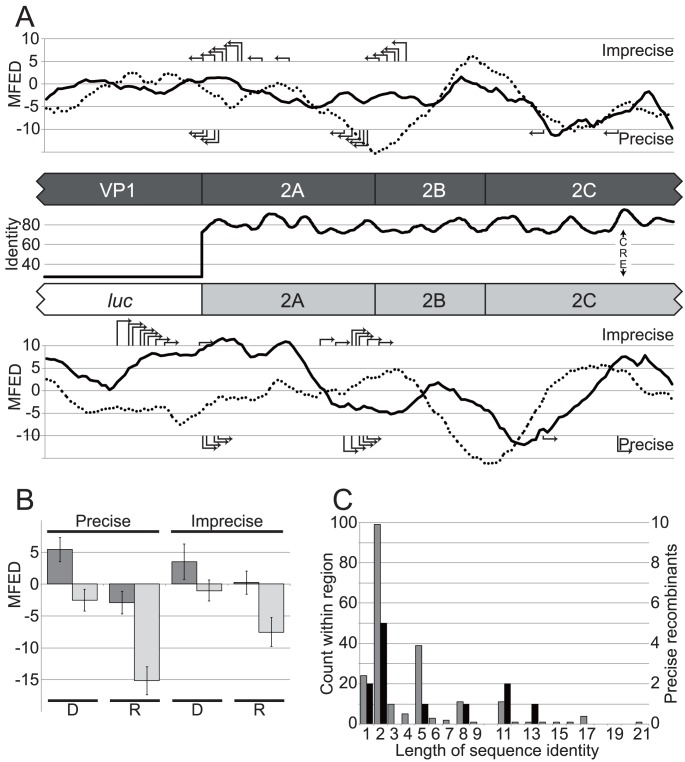
The analysis of recombination junctions. A) Influence of sequence identity and local RNA structure on recombination junctions. The central panel shows the donor (dark shading) and recipient (light shading) partial genomes with protein coding regions indicated flanking a line graph of sequence identity (%) between the non-structural regions of poliovirus type 1 and type 3. Above and below are shown graphs of the mean folding energy differences (MFED; indicated as the percentage difference from 999 sequence-order randomized controls) – a measure of sequence-independent localized RNA structure – over a 250 nt. sliding window (in 30 nt. increments) in the sense and antisense genomes, indicated in solid and dotted lines respectively. The locations of precise and imprecise recombination junctions are indicated in the top and bottom panels by right angle arrows. B) The mean folding energy difference (MFED; percentage difference from 999 sequence order randomized controls) of 100 nt. regions spanning precise and imprecise junctions in donor (sub-genomic replicon) and recipient (CRE-mutant) sequences. Dark and pale shaded bars indicate the MFED of the positive- or negative-sense strand respectively. D and R indicate the donor and recipient sequences respectively. The error bar indicates the standard error. C) Precise junctions do not occur in regions of maximum sequence identity between recombination partners. Individual counts of short lengths of sequence identity between pRLucWT and pT7/SL3 (pale shading; left axis) within the 1058 nt. separating the start of the P2 coding region and the CRE and (dark shading; right axis) counts of precise recombinants exhibiting short regions of identity at the junction (see Figure 3).

Previous studies have implicated local RNA secondary structure, sequence composition or template identity as influencing recombination [64]–[66]. To investigate this further the amount of RNA structure on the positive and negative sense genome was determined by calculating the mean folding energy difference (MFED) between the native sequence and sequence order randomized controls [67] over a sliding 250 nt window spanning the region within which recombination could occur (Figure 5a). MFED values ranged between +5% and −15%, with positive values indicating the presence of sequence order-dependent structure. The average MFED value for sense and antisense strands was 1.58% and −1.56% respectively for the type 1 sub-genomic replicon (the donor genome) and −1.95% and −3.89% respectively for the poliovirus type 3 recipient genome. Neither the precise or imprecise junctions mapped to the regions with maximum or minimum predicted RNA structure (positive and negative values respectively in Figure 5a) between the luciferase coding region and defective CRE. To exclude the possibility that the use of a sliding window during MFED calculations may have obscured limited localized secondary structure, we also determined the MFED value for the 100 nt window spanning each mapped junction in the donor and recipient genomes by comparison of the native sequence to 999 sequence-order randomized controls, using a scrambling algorithm (NDR) that maintained key features, such as dinucleotide composition, of the sequence. Both precise and imprecise junctions exhibited positive MFED (*i.e*. structured) values in the positive strand of donor sequences (Figure 5b; dark bars), though the MFED values were near an arbitrary 4% cutoff we have previously considered marks the lower limit of reliably predicted RNA structure [68]. In contrast, the negative strand (Figure 5b; pale bars) of both precise and imprecise junctions was largely unstructured, and this was particularly marked in the negative strand of precise recipient sequences.

**Figure 6:**
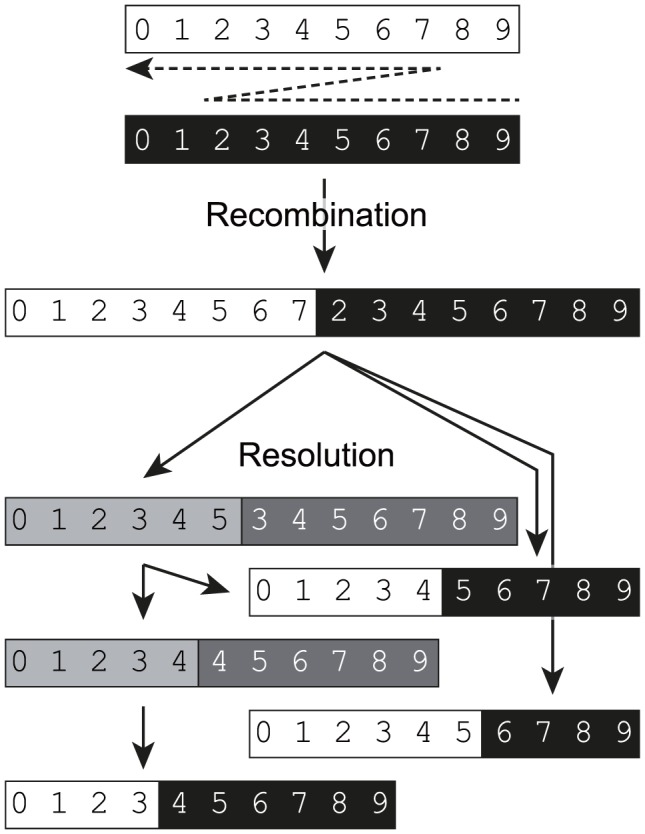
A biphasic model for replicative recombination in enteroviruses. Template switching during negative-strand synthesis (indicated by a dashed arrow) results in the generation of a chimeric intermediate in which parts of the genome are effectively duplicated. Numbering and background shading indicates the source of the original sequences from the parental genomes and shows the duplicated sequences. During subsequent rounds of replication these undergo resolution by internal deletion (or potentially further recombination) events to generate a range of genome-length progeny either directly or via intermediates (indicated by grey background shading). A single initial recombinant can give rise to a range of different genome-length final products. The recombination and resolution events may occur in the initial dually infected cell (in which case negating the requirement for the original chimeric genome to be encapsidated) but – as in the assay presented in Figure 1 – can also occur in a different cell following re-infection.

Sequence identity within the potential recombination region ranged from ∼27% (luciferase and VP1) to almost 98% in the sequence that forms the CRE, and averaged 79.9% within P2-coding region (Figure 6a), distributed as short regions of identity between 2 and 21 nt. in length interspersed with variant nucleotides. There was no correlation between the length of sequence identity and the frequency or distribution of the precise recombination junctions. For example, in the aligned sequences there were 99 distinct conserved dinucleotides (almost all being the first two nucleotides of aligned codons) but only 10 positions with identity of 12 nt. or greater. Of the precise junctions characterised, 50% occurred at positions with identical dinucleotides, with only 10% occurring at regions of identity of more than 12 nt. (Figure 5c, Figure S1). Finally, detailed analysis of the 15 nt. sequences flanking every junction obtained, both precise and imprecise, failed to detect any significant biases whether quantified as individual nucleotides (A,C,G,U), grouped as (A+U) or (C+G) or as purine/pyrimidines (data not shown).

## Discussion

The rapid evolution of single-stranded positive-sense RNA viruses can be attributed to their error-prone RNA dependent RNA polymerases (RdRp) and the ability of their modular genomes to undergo recombination. Coupled with their generally short replication cycles and high yields this allows rapid adaptation to altered environmental conditions and the acquisition of novel tissue or host tropisms [69], [70]. In recipients of the live attenuated poliovirus vaccine recombinants are excreted within 7 days [18], [19], [71]. Furthermore, recombination between Sabin vaccine strains and co-circulating – and co-infecting - enteroviruses can generate novel neurovirulent chimeras capable of spreading within a community with low vaccine coverage [27], [72], [73]. Further studies have demonstrated that recombinant forms of particular serotypes of human enteroviruses wax and wane in their relative geographical dominance with characteristic half lives [23]–[25].

Despite recombination being a well defined phenomena in picornaviruses of humans and animals [13], [74], as well as other important positive-strand RNA virus pathogens [44], [75]–[77], the underlying mechanism(s) by which recombinants arise are relatively poorly understood. Studies have suggested that the copy-choice template-switching model originally proposed [47] was additionally influenced by sequence identity between parental genomes, in particular short direct sequence repeats [65], [78] or by the RNA secondary structure of donor or recipient molecules [49], [66], [79], [80].

These analyses have generally assumed that the selected recombinant genome contains a junction that reflects the point at which the polymerase switched from donor to recipient template. To test this we reasoned it would be necessary to isolate recombinants as soon as possible after they had arisen, before additional selection by serial passage and inevitable competition amongst the viral population. To achieve this we investigated the recombination between a poliovirus sub-genomic replicon [81], [82] and a genome defective in positive-strand RNA synthesis due to disruption of the CRE [55], [57], [83], [84]. By transfecting RNA into rodent cells, which lack the receptor for poliovirus, we prevented reinfection and so could analyse early products of recombination. To ensure we were analyzing replication-competent genomes we biologically cloned progeny virus by limit dilution in HeLa cells before genome analysis.

Having determined that neither parental genome generated viable revertants (Figure 1c) we quantified progeny virus generated by intra- (poliovirus type 3) and inter-serotypic (poliovirus type 1 and type 3) recombination. Using standardized conditions intra-serotypic recombinants arose approximately ten-fold more frequently that inter-serotypic recombinants (Figure 1d). Based upon the known recovery (pfu/µg) of type 1 and type 3 poliovirus in L929 cells following RNA transfection (data not shown) it was estimated that intra-serotypic recombinants would represent ∼1-10% of the progeny, broadly in line with figures previously reported [47], [85], [86]. However, as this would be influenced by absolute levels of replication of the unmodified genomes, by the co-transfection efficiency and by the amount of replication post-recombination, this was not further investigated.

The majority of intratypic poliovirus recombinants were indistinguishable from a parental poliovirus type 3 genome. However, intriguingly, two of the sequences contained genome duplications, of 27 or 78 nt. (Figure 2a). This prompted us to analyse an extensive panel of intertypic poliovirus type 1/type 3 recombinants in which the 21% sequence divergence between the selection markers (the luciferase coding region and the mutated CRE) facilitated identification of recombination junctions. Of 136 genomes in which the sequence could be unambiguously identified, 95 (70%) contained additional sequences, forming 17/20 (85%) distinct sequence junctions analysed. We termed these imprecise junctions to distinguish them from genomes bearing no additional sequences (precise junctions). We prefer the use of the terms precise or imprecise – rather than homologous or non-homologous – as they define the characteristics of the recombination junction with regard to the parental virus genome, rather than the mechanism by which recombination occurred.

Recombinant virus progeny were obtained upon co-transfection of rodent (mouse, hamster) and human (HeLa) cell lines, with no strong evidence for cell specificity; we regularly observed a ∼2.5 fold higher yield in hamster cells compared with mouse cells but consider this reflects differences in transfection efficiency and genome replication (data not shown). Both precise and imprecise recombinants were recovered in all the cell types tested. In rodent cells we observed different ratios of precise to imprecise recombinants in repeated parallel co-transfections. We believe this represents the stochastic temporal nature of an individual recombination event and subsequent replication; recombinants arising soon after transfection would be expected to yield genomes that would undergo multiple additional rounds of replication, with the possibility of secondary events (see below) therefore being more likely to yield precise recombinant progeny with enhanced fitness. It should be noted that the CRE-REP assay we describe here involves analysis of progeny 24-48 hours post transfection. During this period, selection for genomes with enhanced replication likely occurs, so the genomes analysed are an indicative, rather than exhaustive, representation of early recombination products. Based upon our demonstration of enhanced replication kinetics after passage (Figure 3ab) we speculate that precise recombinants arise following rapid selection from an initial imprecise recombinant, and that they quickly outcompete the less fit recombinants to prevail in the analysed population. We also note that precise recombinants were usually represented multiple times during the group analysis (Figure S1), suggesting that once they had appeared in the population their enhanced replication meant that they rapidly became the dominant virus present. This interpretation could be addressed by next generation sequencing analysis of the viral RNA population of co-transfected (or infected) cells in future studies.

All recombinants were harvested from transfected cell supernatant and isolated by limit dilution in HeLa cells. This would have imposed a minimal viability criteria on the genome of being encapsidated and capable of being both translated and replicated. By definition, the imprecise recombinants obtained contained partial duplications of parts of the genome. Although the packaging limit of poliovirus is not known, previously constructed dicistronic viruses have contained at least an additional 573 nt. [87] and the genome of foot and mouth disease virus, which possesses a similarly sized particle, is 8.2 kb. It is therefore unlikely that the largest imprecise genome obtained (#67B; 321 nt.; Figure S1) represents any sort of packaging limit, but rather reflects the selection assay used or the mechanism of recombinant generation. Furthermore, since recombinant populations are usually mixtures (generated *in vivo* in the presence of fully viable parental viruses), both the size and range of the additional sequences may represent genomes which retain sufficient replicative fitness to compete in a mixed infection. How do imprecise recombinants, possibly of lower fitness, compete at all in a natural infection? We think this reflects the stochastic nature of the infection and transmission of enteroviruses. A replication-competent recombinant, even of only limited fitness, shed from the initial dually infected cell would presumably either infect another cell in the same host, or be shed into the environment. Although locally the multiplicity of infection in the original host may be high (and the recombinant poorly, if at all, competitive with parental viruses), transmission to distant sites in the same host or another host may result in a founder effect, in which the recombinant – or subsequent resolved derivatives of it – can proliferate in the absence of parental genomes of potentially greater fitness.

A striking feature of the imprecise recombinants recovered was their clustering in regions encoding the amino and carboxyl termini of 2A^pro^ (Figures 2b, 3d). The consequences of this clustering was that the majority (94/95 individual sequences in 16/17 groups) of imprecise recombinants retained the ability to encode non-chimeric versions of the 2A^pro^ and 2B proteins; the sole exception being #9C in which the additional 3 nt. at the junction occurred within the first few conserved codons of the 2B coding region (Figure S1). The junctions generated in Cluster 1 encoded both 2A^pro^ and 2B from the sub-genomic replicon recombination partner (the polymerase donor) and those in Cluster 2 encoded 2B from the donor and 2A^pro^ from the recipient poliovirus type 3 genome. We propose that this clustering is a consequence of the overriding requirement for functional 2A^pro^ and 2B proteins in the initial recombinant and that this is rarely, if ever, achieved in a single step. Since imprecise recombinants are viable and can serve as the source for subsequent precise recombinants (Figure 3; Figure S2), it seems logical that this mechanism increases the chance by as much as two orders of magnitude (*cf*. generation of a precise recombinant with an imprecise recombinant containing 3–321 nt. of additional sequence) of generating the latter. It should be noted that the polyprotein encoded by imprecise recombinants would include an additional 2A^pro^ or 3C^pro^ cleavage site (Figure 2B). Since these provide an authentic context for proteolytic processing we presume this contributes to the viability of such imprecise recombinants. The 16 nt. insert derived from the 5′ NCR present in #25A must have arisen by two relocations of the viral polymerase with regard to the template. Since this sequence is identical in the parental genomes it is not clear which it was derived from, though at least one of the polymerase template reassociations must have been *in cis*. It is unclear why imprecise recombinants spanning the 2B/2C junction were not recovered in our analysis, whereas precise recombinants in 2C (#4E and #44B; Figure 2b, Figure S1) did occur. One possibility is that the assay used (Figure 1) requires that the hybrid genome is capable of establishing a productive infection in HeLa cells and that imprecise recombinants across 2B/2C are unable to do this due to deficiencies in homodimerisation, membrane permeabilization or formation of the replication complex [88]–[91]. Alternatively, this may indicate that the higher order structure of one or both of these proteins involves a ‘head to tail’ interaction in which the interface is between the amino terminus of one subunit and the carboxyl terminus of the other. Related to this point, it is also interesting to note that there is a reported asymmetry in reciprocal recombinants in the P2 region of poliovirus and related species C coxsackie A viruses which may reflect important protein-protein interactions required for genome replication and particle assembly [45].

The yield of intra-serotype recombinants was ∼10-fold higher than inter-serotype recombinants (Figure 1d) and, although only a limited number were sampled, a greater proportion of the former were precise (87% 13/15 *cf*. 30% 41/136 of inter-serotype recombinants). Further studies will be needed to determine whether the increased proportion of precise junctions is due to the sequence identity of the parental genomes, reflecting its role in the underlying recombination mechanism. This may also influence the yield of recombinants. However, protein-protein compatibility is also likely to influence both the yield and type of viable recombinants generated. Identical parental genomes inevitably encode proteins that have co-evolved and have presumably achieved optimal compatibility. This may enhance the replication and subsequent resolution (see below) of imprecise intra-serotype recombinants. In contrast, even the limited sequence divergence at the amino acid level between the parental serotypes (4–9% divergence at the amino acid level between poliovirus type 1 and 3 proteins 2A^pro^, 2B and 2C), might compromise the fitness of inter-serotype recombinants. Future analysis should therefore include looking for adaptive changes potentially some distance from the recombination site.

We propose that the recombination process of enteroviruses is biphasic (Figure 6), involving the generation of an initial imprecise recombinant which – through a process we term *resolution* – yields precise, genome-length, recombinants. The resolution process may be direct, generating precise recombinants in a single step, or indirect in which imprecise recombinants with shorter genome duplications are generated. For example, the synthesised #105B recombinant cDNA generated both precise and imprecise recombinants upon serial passage (Figure S2). We predict that resolution is an iterative process in which genomes with incrementally increased fitness are selected from the pool of molecules generated during replication. Whether resolution involves intra- or intermolecular polymerase transfer remains to be determined. In practice the resolution event may occur in the same dually infected cell in which the initial recombinant was generated. We think that this is the likely explanation for the generation of precise recombinants in co-transfected L929 murine cells (Figure 2). A biphasic recombination process is likely to have a bearing upon the recombination junctions generated. For example, the processes involved in the generation of imprecise and precise junctions may be influenced – if at all – by different contextual and sequence-dependent attributes. Prompted by the observation that the majority of precise recombinants occur at positions with limited sequence identity between templates (underlined in Figures S1 and S2) we investigated the role of primary sequence and RNA secondary structure. Other than an apparent absence of structure in the negative strand of recipient precise junctions (Figure 6b), which mechanistically seems unlikely to contribute to the polymerase strand-transfer reaction, we found no compelling evidence for a role for either primary sequence or sequence order-dependent RNA structure in influencing the recombination junctions observed. In contrast, Runckel and colleagues have recently reported an elegant next generation sequencing study of precise recombinants of poliovirus partners tagged with synonymous mutations [64] in which sequence composition and RNA structure correlated with, in a predictable and modifiable manner, recombination hotspots. The application of this type of approach to the imprecise junctions that predominate in early recombinant populations is likely to allow the separation of sequence/structural influences on the recombination process *per se* and the subsequent resolution process. The independent isolation of the same precise recombinant by serial passage of #105B virus recovered from co-transfection and from an engineered cDNA (#105B-a and #105B-1; Figure S2) suggests there may to be sequence-dependent influences on the sites of recombination during the process of resolution. Alternatively, this particular recombinant may have a subtle fitness advantage over others generated, thereby prevailing upon serial passage. Further studies on the relative fitness of precise recombinants, for example by competition studies between known levels of two or more input viruses [92], may help elucidate this.

As a prelude to investigating the influence of the polymerase on the generation of recombinants we compared the relative yield of viable recombinants in the CRE-REP assay and a non-replicative assay [50], [51] primed with the same amounts of truncated variants of the same template RNA (Figure 4a). Under these conditions an intratypic CRE-REP assay generated ∼25 fold more recombinants. We went on to conduct three related studies that indicate that the generation of recombinants in the CRE-REP assay is a replicative process. By increasing (ribavirin) or decreasing (G64S) the error rate of the viral polymerase we respectively enhanced or suppressed the recovery of recombinants (Figure 4bd) in the CRE-REP assay. In contrast, neither ribavirin nor the G64S polymerase mutation influenced the yield of recombinants using a non-replicative assay consisting of truncated RNA templates (Figure 4ce). Further support for the replicative generation of recombinants in the CRE-REP assay was provided by the demonstration that nocodazole reduced the yield of recombinants by ∼70%, but had no effect on either genome replication ([62] and Figure S5) or on the yield of recombinants from the non-replicative assay (Figure 4fg).

The three-fold enhancement of poliovirus recombinant recovery in the presence of ribavirin may have implications for the therapeutic use of this antiviral drug. However, we acknowledge that in hepatitis C patients – in which ribavirin is widely used therapeutically – recombination is considered a relatively rare event [93], [94]. A mechanistic exploration of the enhanced recombination observed in the presence of ribavirin clearly deserves further analysis. In preliminary studies we have observed that 5-fluorouracil also enhances the yield of recombinants in the CRE-REP assay (data not shown). It is likely that extension of these mutagenic inhibitor studies will provide further insight into the precise mechanism of template disengagement and re-engagement by the viral polymerase. Indeed, previous studies have already proposed that mismatches induced by mutagens enhance the dissociation of the template and the polymerase [48], [95]. However, it is interesting to note that in a limited range of imprecise recombinants generated in the presence of ribavirin, nucleotide substitutions were not observed at the recombination junctions sequenced (Woodman and Evans, in preparation).

Since the templates used in the non-replicative assay were identical (other than being either 5′ or 3′ truncated) to those used in the CRE-REP assay, recombination must have had to occur between the same genetic markers – the luciferase reporter gene and the defective CRE (Figure 1) – to generate viable progeny. It is therefore interesting to note that non-replicative recombination generated only ∼4% of the progeny of that produced in the equivalent CRE-REP assay when transfecting equimolar amounts of *in vitro* synthesized RNA into L929 cells (Figure 6a). This striking difference in yield is further support that the CRE-REP assay is likely mechanistically different from a non-replicative recombination event. Further support for this interpretation includes the lack of inhibition of non-replicative recombination by nocodazole – this implies this process occurs outside the confines of the replication complex, or at least does not require co-occupied replication complexes to occur. Finally, although non-replicative recombination, presumably involving cellular RNA exonucleases and ligases, is well documented in a number of picornaviruses, flaviviruses and alphaviruses [51]–[53], [96] – and may generate the types of junctions we define as imprecise [53] – the relative importance *in vivo* of replicative and non-replicative mechanisms of recombinant generation remain to be determined.

The CRE-REP assay we describe may also provide an approach to study the restrictions on intra- and interspecific recombination in enteroviruses, the former being far more frequent than the latter. In preliminary studies we have recovered recombinants between species B enteroviruses (echovirus 7; data not shown), suggesting that the mechanism proposed from our studies using poliovirus is likely generic to other enteroviruses, and presumably to other picornaviruses which also exhibit recombination [97]. However, in repeated attempts we have been unable to recover interspecies recombinants between reciprocal transfections of species C and species B enteroviruses (poliovirus type 1 and either echovirus types 6 or 7 respectively; data not shown). Further studies may elucidate whether this is due to direct genomic incompatibility, *i.e*. any recombinant genomes generated are non-viable, perhaps because they are unable to replicate or undergo proteolytic processing, or due to the absence of protein-protein interactions necessary for encapsidation [46]. Alternatively the rarity of interspecies recombination may reflect a lack of opportunity, perhaps due to occupancy of distinct replication complexes, sub-cellular or cellular compartmentalization.

The suggestion that an imprecise replicative recombination process results in partial duplication of the virus genome also has intriguing implications for our wider understanding of the evolution of positive strand RNA viruses. There are several variously well characterised evolutionary duplication events that have shaped extant picornavirus genomes. These include the capsid proteins (VP1-3) which share a common ‘jelly roll’ structure [98], the three contiguous VPg proteins of foot and mouth disease virus [99] and even the non-contiguous 2A and 3C proteases [100]. In addition, other positive strand RNA viruses exhibit genome duplications [101] such as the multiple VPg proteins of some dicistroviruses [102] or the functionally distinct adjacent leader proteinases of the *Closteroviridae* [103]. The type of imprecise recombination event we describe, potentially coupled with partial resolution, could account for this ‘evolution by duplication’. Likewise, duplication and subsequent resolution events could explain the permutation of the polymerase palm domain reported in some Alphavirus-like insect tetraviruses [104]. Other than the ancestral evolution of the capsid proteins the majority of these involve duplication of relatively short sequences – this is presumed to reflect intrinsic constraints on genome length [101], but may also reflect the mechanism by which they are generated.

An improved insight into evolution of RNA viruses, including acquisition of extensive regions of the genome by the process of recombination, will improve our understanding of the basic biology of this important group of viruses and help us identify how the process can be controlled, avoided or exploited.

## Materials and Methods

### Virus and cell culture

HeLa, L929 murine fibroblasts and baby hamster kidney (BSR-T7) cells were grown as monolayers in Dulbecco's Modified Eagle Medium (DMEM) or Glasgow Minimum Essential Medium (GMEM supplemented with G418 antibiotic). Media was supplemented with 100 U/ml penicillin, 100 µg/ml streptomycin, 2 mM L-glutamine and 10% Heat Inactivated (HI)-FBS. All cells were passaged in the presence of trypsin-EDTA. Where stated, ribavirin (Sigma) or guanidine hydrochloride (Sigma) were added to growth media at the concentrations indicated. Nocodazole was used where required by treating pre-chilled cells (10 minutes at 0°C) with a final concentration of 5 µM in DMSO (essentially as described previously; [62]) for two hours prior to transfection. Poliovirus type 1 (Mahoney) and type 3 (Leon) were recovered following transfection of RNA generated *in vitro* (see below) from full length cDNA. Virus was quantified by plaque assay as described previously [105]. Growth kinetics of viruses were determined following synchronous infection of HeLa cells at a multiplicity of infection (moi) of 10 pfu/cell. Unabsorbed virus was removed by washing with sterile PBS and plates incubated in fresh media at 37°C in an atmosphere containing 5% CO2. Virus in the supernatant was quantified at various time points post infection by plaque assay. Virus competition assays were conducted by co-infection of HeLa cells with an moi of 10 pfu/cell of each virus, removing unabsorbed virus by washing in PBS and harvest of virus from the fresh supernatant at either 24 hours post infection (p.i.) or when cells displayed at least 50% cytopathic effect (cpe). When serially passaging virus, harvested supernatant was diluted 1∶4 with fresh media. Recombinant viruses were biologically cloned by limit (doubling) dilution in 96-well plates seeded with 1×10^5^ HeLa cells/well. Plates were inoculated and incubated at 37°C/5% CO_2_ for four days after which time virus-containing supernatant was removed (and stored) and the remaining cell monolayer stained with crystal violet. Virus supernatant from the highest dilution causing complete cpe was retained for further analysis.

### Plasmids, *in vitro* transcription and cell transfection

pT7Rep3-L is a poliovirus type 3 (Leon) sub-genomic replicon bearing a luciferase reporter gene inserted in-frame in place of the P1 capsid coding region of the genome. pRLucWT is a pBR-based plasmid containing a cDNA for a poliovirus type 1 sub-genomic replicon in which the capsid coding region is replaced, in frame, with a luciferase reporter gene, as previously described [106]. pT7/SL3 has also been described previously [56] and consists of a full-length poliovirus type 3 (Leon) cDNA bearing 8 synonymous substitutions in the *cis*-acting replication element (CRE) within the 2C-coding region. A variant of pRLucWT containing a Gly to Ser substitution at residue 64 (hence designated pRLucWT_G64S_) of the RNA dependent RNA polymerase was generously provided by Craig Cameron and Jamie Arnold. The same mutation was built into the pT7/SL3 cDNA using standard methods. Plasmids pT7/SL3 and pRLucWT were linearised with *Sal* I and *Apa* I respectively and transcribed *in vitro* using T7 RNA Polymerase (Fermentas) following the manufacturer's protocol. Residual DNA template was removed by addition of 2 U DNase Turbo (Ambion) and the RNA transcripts were purified using RNeasy Mini Kit (Qiagen) before spectrophotometric quantification. Unless otherwise stated, 0.5 µg or 1 µg (total) of equimolar amounts of both template RNAs was prepared with Lipofectamine 2000 (Invitrogen; used according to the manufacturers instructions) and transfected into confluent monolayers in 12 or 6 well dishes respectively.

A full length cDNA of the recombinant virus genome designated #105B was constructed using standard molecular biology techniques from the parental pRLucWT and pT7/SL3 cDNAs together with the central region of the genome which was RT-PCR amplified from #105B virus. The compete cDNA sequence was validated before further analysis.

Truncated cDNA templates were generated from pT7Rep3-L, pRLucWT and pT7/SL3 for use in non-replicative recombination assays, essentially as previously described [50]. Briefly, pT7Rep3-L and pRLucWT were digested with *Pml* I and *Pac* I and religated, effectively removing the entire IRES and the majority of the luciferase coding region. Similarly, pT7/SL3 was digested with *Xho* I and *Sal* I and religated, removing the polymerase coding region. Truncated templates are indicated with a prefix or suffix Δ where appropriate *e.g*. pT7/SL3Δ. Where required, template cDNAs were modified by inclusion of a G64S substitution in the viral polymerase using standard methods. Non-replicative recombination assays were always conducted in parallel with the CRE-REP assay (see above) and used equimolar ratios of RNA (total 0.5 µg) co-transfected into L929 murine cells in 12 well dishes. Supernatant virus was recovered, 60 hours post-transfection, and quantified by plaque assay on HeLa cell monolayers.

### Oligonucleotides, viral RNA isolation and characterisation

Viral RNA was extracted from clarified culture supernatant using a Qiagen RNAeasy Mini kit, reverse transcribed using Superscript II reverse transcriptase (Invitrogen) and an oligo-dT primer at 46°C for 50 minutes (mins.) with the reaction terminated by incubation for 15 mins. at 70°C. PCR amplification of recombination junctions used template cDNA and appropriate oligonucleotides as listed in the results section (all oligonucleotides are listed in Supplementary Table S1) with KOD XL DNA polymerase (Novagen) used according to the manufacturer's protocol. PCR products were sized by agarose gel electrophoresis and sequenced by the University of Warwick Genomics Facility on an ABI PRISM 3130xl Genetic Analyser. Sequence analysis used a combination of Lasergene v.6.0 (DNA*) and Clustal [107] as appropriate.

### Nomenclature for recombinant identification

Reference sequences of poliovirus type 3 Leon (Genbank #X00925), type 1 Mahoney (Genbank #V01149) or the type 1 Mahoney-derived luciferase-encoding sub-genomic replicon (pRLucWT). To facilitate definition of junctions defined in clonal groups of recombinant virus genomes a standardized naming scheme was used; the 5′ and 3′ components were numbered with the last or first nucleotide of the relevant parental genome (poliovirus type 3 or the type 1 derived sub-genomic replicon). For reference, the latter consists of a 5′ untranslated region (nucleotides 1–742), the luciferase encoding reporter gene (nt. 743–2410) fused with a short linker to the sequence encoding the last 5 amino acids of VP1. In this cDNA, nonstructural protein coding region starts at nt. 2441. In cases where the recombination junction could not be unambiguously defined – due to sequence identity between aligned parental genomes – the numbering assumes these sequences were derived from the parental genome contributing the 5′ portion of the genome. For example, PV3^3897^/PV1^2737^ (recombination group #14C; Figure 3) consists of nucleotides 1-3897 from PV3 and 2737-polyA from the sub-genomic replicon, pRLucWT.

### Bioinformatic analysis

Mean folding energy differences were calculated essentially as described previously [67] using a sequence scrambling algorithm (NDR) that retains compositional features such as the dinucleotide frequencies of the native sequence as implemented in the SSE package [108].

## Supporting Information

Figure S1 Analysis of heterotypic recombination junctions. Sequence junctions in the 20 distinct heterotypic recombinant groups (prefixed #) identified. Upper and lower case sequences are used to distinguish between those derived from the poliovirus type 3 CRE-defective genome (pT7/SL3) and poliovirus type 1 sub-genomic replicon (pRLucWT) respectively, with the extent of the sequence numbered according to the reference genomes (see Materials and Methods). Sequences derived from the region encoding the luciferase reporter gene indicated by lower case **bold text**. Numbers in parentheses indicate the total number of independent clones of this sequence obtained. The extent of additional sequences at the recombination junction are indicated to the right of each sequence, prefixed with a plus sign. In group #25A sequences derived from the 5′ NCR, nucleotides 447–462 (identical in both parental viruses), are highlighted as white text on a black background. **Calculation of the length of duplicated sequences in imprecise recombinants**. The poliovirus type 1 sub-genomic replicon (pRLucWT) contains a luciferase reporter gene in place of the P1 coding region. For Cluster 1 recombinants it is necessary to determine the length of the additional luciferase sequences present plus the distance from the start of the P2 coding region to the position in the aligned poliovirus type 1 and type 3 sequences. For example, #105B consists of nucleotides 1–3371 inclusive of poliovirus type 3, joined to nucleotide 2287 from the sub-genomic replicon. In the latter the luciferase coding region extends to nucleotide 2440, with nucleotide 2441 being the start of the P2 coding region (which corresponds to nucleotide 3386 in the reference poliovirus type 1 sequence). A numbered alignment of the P2 coding regions is presented in Figure S6. The position – in poliovirus type 1 – in this alignment corresponding to nucleotide 3372 of poliovirus type 3 is 3481. Therefore the length of the additional sequence in #105B is 249 nt, made up of 154 nt. of luciferase and 95 nt. of the first part of the P2 coding region of poliovirus type 1. Similarly, the length of additional sequences present in Cluster 2 imprecise recombinants can be determined from the alignment presented in Figure S6. For example, #9C consists of nucleotides 1–3830 of poliovirus type 3 then from nucleotide 2892 of the poliovirus type 1 sub-genomic replicon to the end of the genome. Position 2892 corresponds to nucleotide 3827 of the reference poliovirus type 1 sequence. By reference to the alignment of the P2 region of poliovirus type 1 and type 3 (Figure S6) it can be determined that #9C contains 3 additional nucleotides at the imprecise junction.
(EPS)

Figure S2 Sequence analysis of cluster 1 (#105B, #53A) and cluster 2 (#51G, #E1) imprecise recombinants after serial passage in HeLa cells. Sequences are numbered as indicated in Figure S1. Sequences with a numeric suffix (*i.e*. #105B-1 to -3) were generated after serial passage of virus derived from a constructed full length #105B cDNA (see text for details).
(EPS)

Figure S3 Ribavirin does not influence the transfection efficiency of L929 murine cells. Ribavirin at the concentrations indicated was included in the supernatant media for two hours pre-transfection and during all subsequent analysis. The poliovirus type 3 sub-genomic replicon pT7Rep3-L was linearized with *Xho* I (which cleaves the cDNA at nt. 6050 within the P3 coding region) and used as a template for T7 polymerase-mediated *in vitro* RNA synthesis. 250 ng of RNA was transfected into L929 cell monolayers in a 12-well microplate and luciferase activity quantified (see Materials and Methods) 4 hours post-transfection. The figures plotted indicate the average of three samples, with the standard deviation shown as error bars.
(PDF)

Figure S4 A G64S high-fidelity polymerase mutation does not inhibit poliovirus replication. Murine L929 cells were transfected with 250 ng of *in vitro* synthesised RNA generated from pRLucWT or pRLucWT_G64S_ linearized with *Apa* I. Encoded luciferase was quantified at 2.5, 5 and 7.5 hours post-transfection. Error bars indicate the standard deviation of two independent samples.
(PDF)

Figure S5 Nocodazole does not influence the yield of poliovirus. Murine L929 cells were chilled and treated with nocodazole (see Materials and Methods) or treated with carrier (DMSO) alone, transfected with 250 ng per well (12 well plate) of *in vitro* synthesised RNA from a full length infectious cDNA of poliovirus type 3 and virus yield at 48 hours post-transfection quantified by plaque assay. Error bars indicate the standard deviation of three independent samples.
(PDF)

Figure S6 CLUSTAL 2.1 multiple sequence alignment of the P2 and P3 coding regions of poliovirus type 1 and poliovirus type 3. * indicates identity. Numbering indicates the position in the reference sequences: Poliovirus type 3 Leon (Genbank #X00925) and Poliovirus type 1 Mahoney (Genbank #V01149).
(PDF)

Table S1 Oligonucleotide primers used in the study. Degenerate sequences are indicated using standard IUPAC codes.
(PDF)
