# Supplementary figures and images for: Recombination in Enteroviruses Is a Biphasic Replicative Process Involving the Generation of Greater-than Genome Length ‘Imprecise’ Intermediates
Source: PLoS Pathog. 2014 Jun 12;10(6):e1004191. doi: 10.1371/journal.ppat.1004191 (PMC4055744; doi:10.1371/journal.ppat.1004191)

## Transfection efficiency with various levels of ribavirin

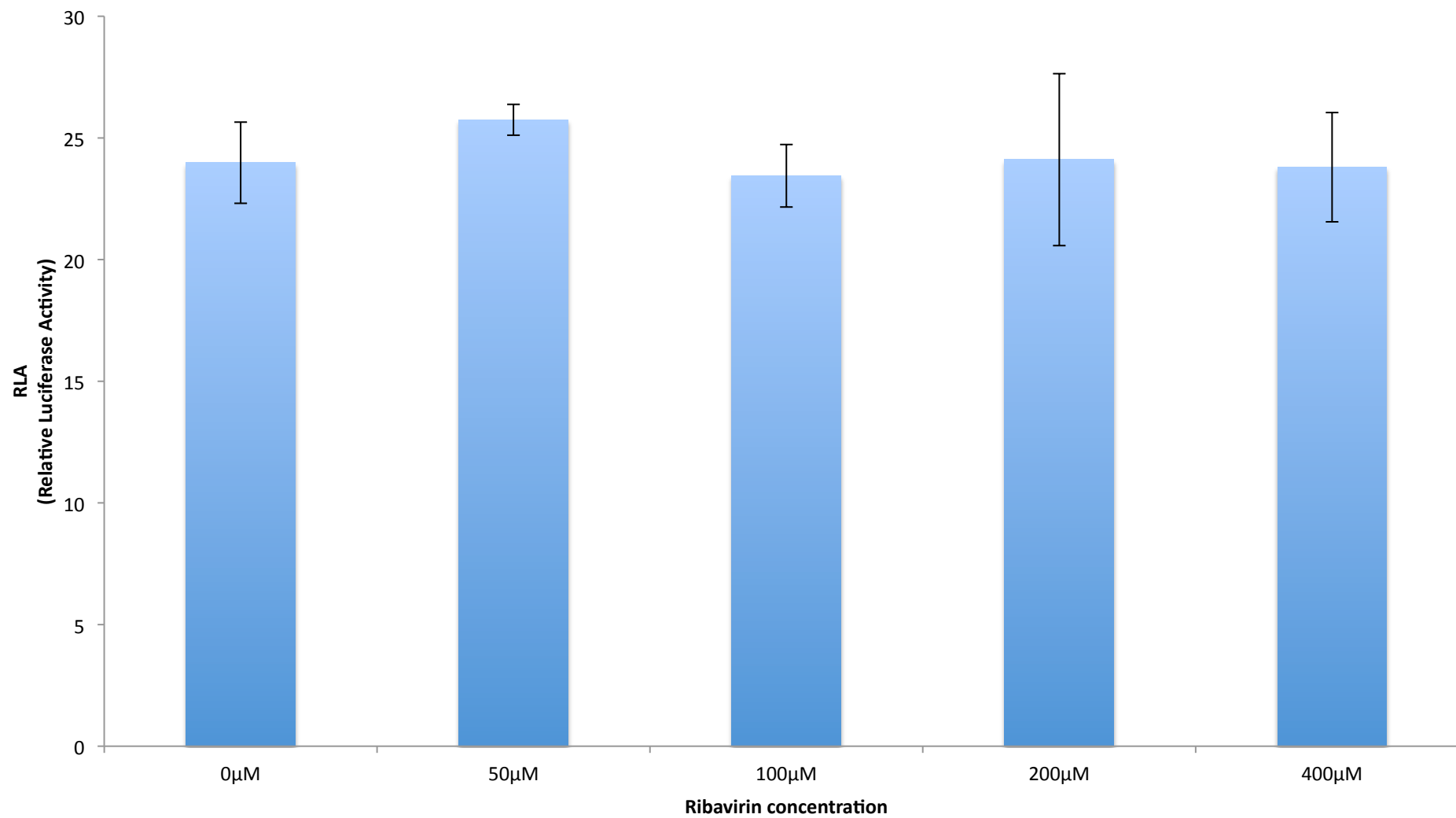

Supplement: Figure S3 — Ribavirin does not influence the transfection efficiency of L929 murine cells. Ribavirin at the concentrations indicated was included in the supernatant media for two hours pre-transfection and during all subsequent analysis. The poliovirus type 3 sub-genomic replicon pT7Rep3-L was linearized with Xho I (which cleaves the cDNA at nt. 6050 within the P3 coding region) and used as a template for T7 polymerase-mediated in vitro RNA synthesis. 250 ng of RNA was transfected into L929 cell monolayers in a 12-well microplate and luciferase activity quantified (see Materials and Methods) 4 hours post-transfection. The figures plotted indicate the average of three samples, with the standard deviation shown as error bars. (PDF) [file ppat.1004191.s003.pdf]

pRLucWT - v - pRLuc-G64S

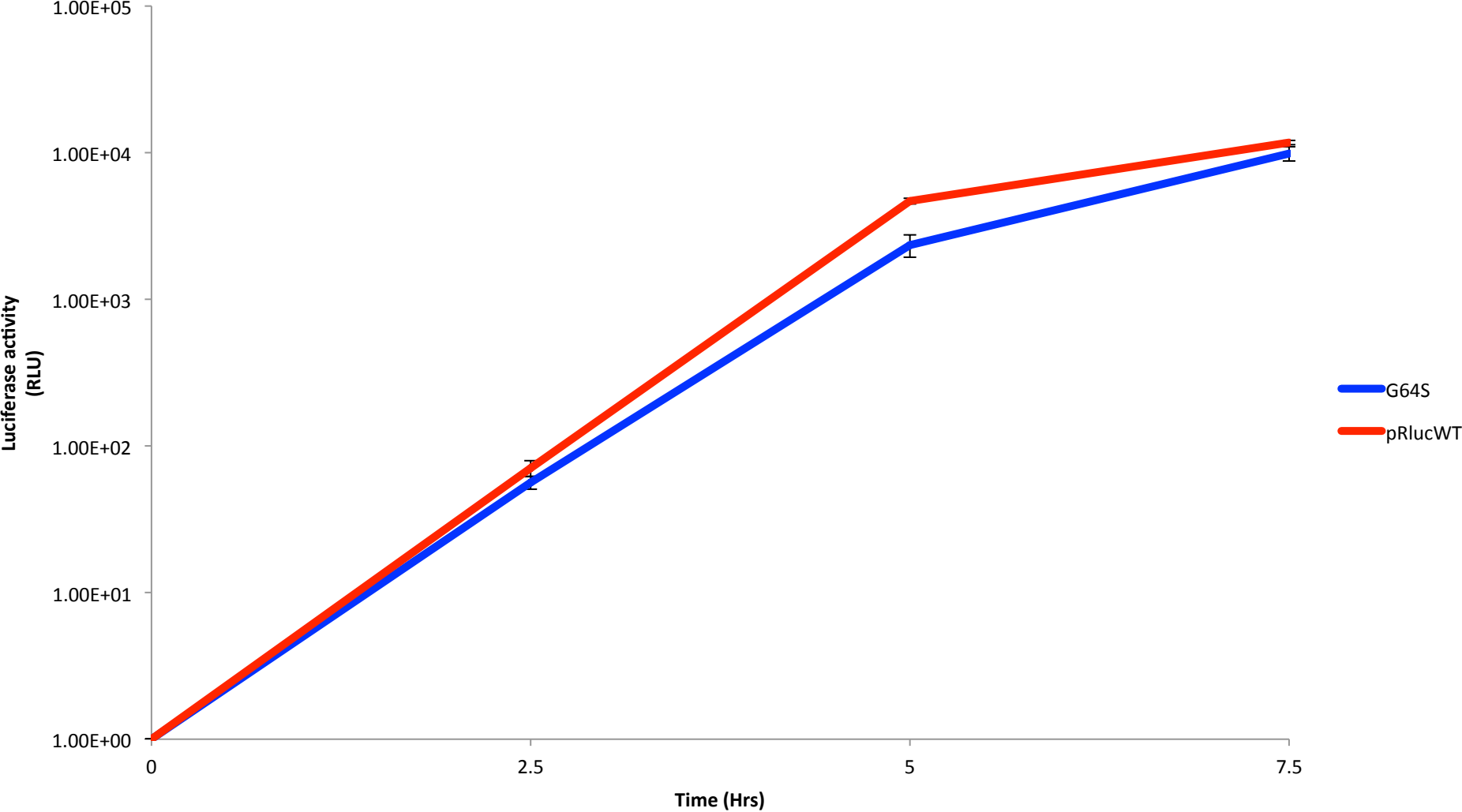

Supplement: Figure S4 — A G64S high-fidelity polymerase mutation does not inhibit poliovirus replication. Murine L929 cells were transfected with 250 ng of in vitro synthesised RNA generated from pRLucWT or pRLucWTG64S linearized with Apa I. Encoded luciferase was quantified at 2.5, 5 and 7.5 hours post-transfection. Error bars indicate the standard deviation of two independent samples. (PDF) [file ppat.1004191.s004.pdf]

## PV3FLC +/- Nocodazole

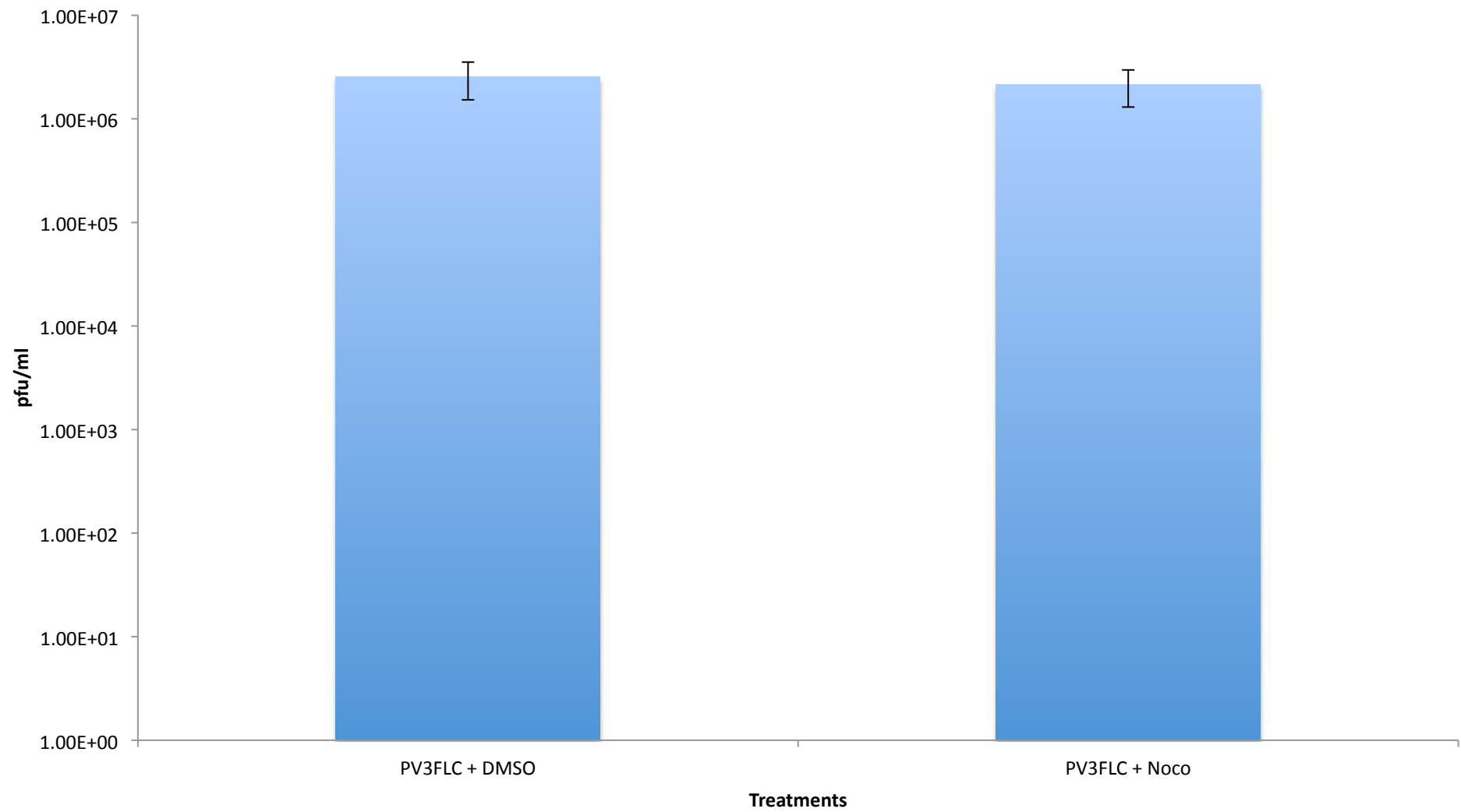

Supplement: Figure S5 — Nocodazole does not influence the yield of poliovirus. Murine L929 cells were chilled and treated with nocodazole (see Materials and Methods) or treated with carrier (DMSO) alone, transfected with 250 ng per well (12 well plate) of in vitro synthesised RNA from a full length infectious cDNA of poliovirus type 3 and virus yield at 48 hours post-transfection quantified by plaque assay. Error bars indicate the standard deviation of three independent samples. (PDF) [file ppat.1004191.s005.pdf]
