# Supplementary material for: Recombination in Enteroviruses Is a Biphasic Replicative Process Involving the Generation of Greater-than Genome Length ‘Imprecise’ Intermediates
Source: PLoS Pathog. 2014 Jun 12;10(6):e1004191. doi: 10.1371/journal.ppat.1004191 (PMC4055744; doi:10.1371/journal.ppat.1004191)
Supplement: Figure S6 — CLUSTAL 2.1 multiple sequence alignment of the P2 and P3 coding regions of poliovirus type 1 and poliovirus type 3. * indicates identity. Numbering indicates the position in the reference sequences: Poliovirus type 3 Leon (Genbank #X00925) and Poliovirus type 1 Mahoney (Genbank #V01149). (PDF) [file ppat.1004191.s006.pdf]

```
PV3                                                                    GGCTT 3381
PV1                                                                    GGATT 3390
                                                                      ** **

PV3             TGGGCATCAGAATAAAGCTGTGTACACTGCTGGTTACAAGATCTGCAACTACCATCTCGC 3441
PV1             CGGACACCAAAACAAAGCGGTGTACACTGCAGGTTACAAAATTTGCAACTACCACTTGGC 3450
                 ** ** ** ** ***** *********** ******** ** ***********  * **

PV3             CACTAAGGAGGATTTACAAAATGCTGTAAGCATCATGTGGAATAGAGACCTCTTGGTTGT 3501
PV1             CACTCAGGATGATTTGCAAAACGCAGTGAACGTCATGTGGAGTAGAGACCTCTTAGTCAC 3510
                **** **** ***** ***** ** ** * * ********* *********** **

PV3             TGAATCAAAAGCTCAAGGTACCGACTCAATAGCAAGGTGCAATTGCAATGCAGGGGTGTA 3561
PV1             AGAATCAAGAGCCCAGGGCACCGATTCAATCGCAAGGTGCAATTGCAACGCAGGGGTGTA 3570
                 ******* *** ** ** ***** ***** ***************** ***********

PV3             CTATTGTGAGTCCAGAAGGAAATACTACCCTGTGTCGTTTGTGGGACCCACCTTCCAATA 3621
PV1             CTACTGCGAGTCTAGAAGGAAATACTACCCAGTATCCTTCGTTGGCCCAACGTTCCAGTA 3630
                *** ** ***** ***************** ** ** ** ** ** ** ** ***** **

PV3             CATGGAGGCTAATGACTACTACCCAGCTAGATACCAATCCCACATGTTAATCGGGCACGG 3681
PV1             CATGGAGGCTAATAACTATTACCCAGCTAGGTACCAGTCCCATATGCTCATTGGCCATGG 3690
                ************* **** *********** ***** ***** *** * ** ** ** **

PV3             CTTTGCCTCACCAGGTGACTGTGGTGGTATCCTTAGGTGTCAACATGGCGTCATCGGAAT 3741
PV1             ATTCGCATCTCCAGGGGATTGTGGTGGCATACTCAGATGTCACCACGGGGTGATAGGGAT 3750
                 ** ** ** ***** ** ******** ** ** ** ***** ** ** ** ** ** **

PV3             CGTGACAGCTGGTGGAGAGGGATTAGTCGCATTCTCTGACATAAGGGACTTGTATGCTTA 3801
PV1             CATTACTGCTGGTGGCGAAGGGTTGGTTGCATTTTCAGACATTAGAGACTTGTATGCCTA 3810
                * * ** ******** ** ** ** ** ***** ** ***** ** *********** **

PV3             CGAGGAAGAGGCCATGGAGCAGGGCATTTCAAACTATATTGAGTCACTCGGTGCTGCGTT 3861
PV1             CGAAGAAGAAGCCATGGAACAAGGCATCACCAATTACATAGAGTCACTTGGGGCCGCATT 3870
                *** ***** ******** ** *****  * ** ** ** ******** ** ** ** **

PV3             CGGTAGTGGGTTCACTCAGCAAATAGGGGATAAGATATCAGAACTAACCAGCATGGTGAC 3921
PV1             TGGAAGTGGATTTACTCAGCAGATTAGCGACAAAATAACAGAGTTGACCAATATGGTGAC 3930
                 ** ***** ** ******** **  * ** ** *** ****  * ****  ********

PV3             CAGCACGATTACAGAGAAGCTACTTAAAAACCTAATCAAAATTATTTCATCTCTGGTGAT 3981
PV1             CAGTACCATCACTGAAAAGCTACTTAAGAACTTGATCAAGATCATATCCTCACTAGTTAT 3990
                *** ** ** ** ** *********** *** * ***** ** ** ** ** ** ** **

PV3             TATCACTAGAAATTACGAAGATACCACCACAGTGCTCGCCACTCTAGCTCTTCTTGGGTG 4041
PV1             TATAACTAGGAACTATGAAGACACCACAACAGTGCTCGCTACCCTGGCCCTTCTTGGGTG 4050
                *** ***** ** ** ***** ***** *********** ** ** ** ***********

PV3             TGATGTTTCACCGTGGCAATGGCTGAAGAAGAAAGCATGTGACACTTTGGAGATTCCCTA 4101
PV1             TGATGCTTCACCATGGCAGTGGCTTAGAAAGAAAGCATGCGATGTTCTGGAGATACCTTA 4110
                ***** ****** ***** ***** *  *********** **   * ******* ** **

PV3             TGTTATTAGACAGGGTGATAGTTGGTTGAAAAAATTTACTGAGGCGTGCAACGCAGCTAA 4161
PV1             TGTCATCAAGCAAGGTGACAGTTGGTTGAAGAAGTTTACTGAAGCATGCAACGCAGCTAA 4170
                *** ** *  ** ***** *********** ** ******** ** **************

PV3             GGGGTTGGAATGGGTGTCCAACAAAATCTCAAAATTTATTGACTGGTTGAGAGAAAGAAT 4221
PV1             GGGACTGGAGTGGGTGTCAAACAAAATCTCAAAATTCATTGATTGGCTCAAGGAGAAAAT 4230
                ***  **** ******** ***************** ***** *** * *  ** * ***

PV3             CATCCCACAAGCCAGGGACAAGCTTGAGTTTGTAACCAAATTGAAACAGTTGGAAATGCT 4281
PV1             TATCCCACAAGCTAGAGATAAGTTGGAATTTGTAACAAAACTTAGACAACTAGAAATGCT 4290
                 *********** ** ** *** * ** ******** *** * * ***  * ********

PV3             AGAGAATCAGATATCCACAATACACCAATCTTGTCCAAGTCAGGAACACCAGGAAATTTT 4341
PV1             GGAAAACCAAATCTCAACTATACACCAATCATGCCCTAGTCAGGAACACCAGGAAATTCT 4350
                 ** ** ** ** ** ** *********** ** ** ********************* *

PV3             GTTCAACAATGTACGCTGGTTGTCCATTCAATCCAAGAGATTCGCTCCATTGTACGCACT 4401
PV1             ATTCAATAATGTCAGATGGTTATCCATCCAGTCTAAGAGGTTTGCCCCTCTTTACGCAGT 4410
                 ***** *****  * ***** ***** ** ** ***** ** ** **  * ****** *

PV3             TGAGGCCAAGAGAATACAAAAGTTGGAACACACCATTAATAATTACATACAGTTCAAGAG 4461
PV1             GGAAGCCAAAAGAATACAGAAACTAGAGCATACTATTAACAACTACATACAGTTCAAGAG 4470
                 ** ***** ******** **  * ** ** ** ***** ** *****************

PV3             CAAACACCGTATTGAGCCAGTATGTTTGTTAGTGCATGGGAGCCCAGGTACAGGAAAATC 4521
PV1             CAAACACCGTATTGAACCAGTATGTTTGCTAGTACATGGCAGCCCCGGAACAGGTAAATC 4530
                *************** ************ **** ***** ***** ** ***** *****

PV3             AGTTGCGACTAACCTAATTGCTAGAGCCATAGCTGAGAAAGAGAACACCTCCACCTACTC 4581
PV1             TGTAGCAACCAACCTGATTGCTAGAGCCATAGCTGAAAGAGAAAACACGTCCACGTACTC 4590
                 ** ** ** ***** ******************** * *** ***** ***** *****

PV3             GCTACCACCGGACCCGTCTCACTTTGATGGATACAAACAACAAGGTGTGGTTATCATGGA 4641
PV1             GCTACCCCCGGATCCATCACACTTCGACGGATACAAACAACAGGGAGTGGTGATTATGGA 4650
                ****** ***** ** ** ***** ** ************** ** ***** ** *****
```

```
PV3             CGACCTAAACCAAAACCCGGATGGGGCAGATATGAAGCTCTTTTGTCAAATGGTGTCCAC 4701
PV1             CGACCTGAATCAAAACCCAGATGGTGCGGACATGAAGCTGTTCTGTCAGATGGTATCAAC 4710
                ****** ** ******** ***** ** ** ******** ** ***** ***** ** **

PV3             TGTGGAGTTTATCCCACCTATGGCCTCGCTGGAAGAGAAAGGCATTCTGTTCACATCCAA 4761
PV1             AGTGGAGTTTATACCACCCATGGCATCCCTGGAGGAGAAAGGAATCCTGTTTACTTCAAA 4770
                 *********** ***** ***** ** ***** ******** ** ***** ** ** **

PV3             CTATGTTTTAGCCTCCACCAACTCCAGTCGCATCACACCACCTACAGTAGCCCACAGTGA 4821
PV1             TTACGTTCTAGCATCCACAAACTCAAGCAGAATTTCCCCCCCCACTGTGGCACACAGTGA 4830
                 ** *** **** ***** ***** **  * **  * ** ** ** ** ** ********

PV3             CGCTCTGGCCAGGAGGTTCGCTTTCGATATGGATATTCAAGTGATGGGCGAGTACTCCAG 4881
PV1             TGCATTAGCCAGGCGCTTTGCGTTCGACATGGACATTCAGGTCATGAATGAGTATTCTAG 4890
                 **  * ****** * ** ** ***** ***** ***** ** ***   ***** ** **

PV3             AGATGGTAAACTCAACATGGCAATGGCTACTGAGACGTGCAAGGACTGCCACCAACCAGC 4941
PV1             AGATGGGAAATTGAACATGGCCATGGCTACTGAAATGTGTAAGAACTGTCACCAACCAGC 4950
                ****** *** * ******** *********** * *** *** **** ***********

PV3             AAACTTCAAAAGATGCTGTCCTTTAGTGTGTGGTAAGGCAATTCAGTTAATGGACAAATC 5001
PV1             AAACTTTAAGAGATGCTGTCCTTTAGTGTGTGGTAAGGCAATTCAATTAATGGACAAATC 5010
                ****** ** ************************************ *************

PV3             TTCCAGAGTTAGGTACAGTGTTGACCAGATTACTACAATGATTATCAACGAGAGAAACAG 5061
PV1             TTCCAGAGTTAGATACAGTATTGACCAGATCACTACAATGATTATCAATGAGAGAAACAG 5070
                ************ ****** ********** ***************** ***********

PV3             AAGATCTAACATTGGCAATTGCATGGAGGCTTTGTTCCAAGGACCACTCCAGTACAAAGA 5121
PV1             AAGATCCAACATTGGCAATTGTATGGAGGCTTTGTTTCAAGGACCACTCCAGTATAAAGA 5130
                ****** ************** ************** ***************** *****

PV3             CCTGAAAATTGACATCAAGACGAGGCCCCCCCCTGAATGCATCAATGATCTGCTTCAAGC 5181
PV1             CTTGAAAATTGACATCAAGACGAGTCCCCCTCCTGAATGTATCAATGACTTGCTCCAAGC 5190
                * ********************** ***** ******** ********  **** *****

PV3             AGTTGACTCCCAGGAAGTGAGGGATTATTGTGAAAAGAAAGGATGGATCGTCAACATCAC 5241
PV1             AGTTGACTCCCAGGAGGTGAGAGATTACTGTGAGAAGAAGGGTTGGATAGTCAACATCAC 5250
                *************** ***** ***** ***** ***** ** ***** ***********

PV3             TAGCCAAGTTCAAACAGAGAGAAACATTAACCGAGCAATGACCATTTTGCAGGCAGTGAC 5301
PV1             CAGCCAGGTTCAAACAGAAAGGAACATCAACAGGGCAATGACAATTCTACAAGCGGTGAC 5310
                 ***** *********** ** ***** *** * ******** *** * ** ** *****

PV3             AACTTTCGCCGCAGTGGCTGGTGTCGTGTACGTCATGTACAAGTTATTCGCTGGACACCA 5361
PV1             AACCTTCGCCGCAGTGGCTGGAGTTGTCTATGTCATGTATAAACTGTTTGCTGGACACCA 5370
                *** ***************** ** ** ** ******** **  * ** ***********

PV3             GGGAGCATACACTGGTCTGCCAAACAAAAGACCCAATGTGCCCACCATTAGAGCAGCAAA 5421
PV1             GGGAGCATACACTGGTTTACCAAACAAAAAACCCAACGTGCCCACCATTCGGACAGCAAA 5430
                **************** * ********** ****** ************ *  *******

PV3             AGTGCAAGGGCCTGGGTTTGACTATGCAGTGGCTATGGCTAAAAGAAACATTGTTACAGC 5481
PV1             GGTACAAGGACCAGGGTTCGATTACGCAGTGGCTATGGCTAAAAGAAACATTGTTACAGC 5490
                 ** ***** ** ***** ** ** ***********************************

PV3             AACTACTAGCAAAGGGGAGTTCACAATGCTAGGAGTCCACGACAACGTGGCCATTTTACC 5541
PV1             AACTACTAGCAAGGGAGAGTTCACTATGTTAGGAGTCCACGACAACGTGGCTATTTTACC 5550
                ************ ** ******** *** ********************** ********

PV3             AACTCATGCCTCACCTGGTGAGAGTATTGTAATTGATGGCAAAGAGGTTGAAATCCTAGA 5601
PV1             AACCCACGCTTCACCTGGTGAAAGCATTGTGATCGATGGCAAAGAAGTGGAGATCTTGGA 5610
                *** ** ** *********** ** ***** ** *********** ** ** *** * **

PV3             CGCTAAAGCCCTCGAAGATCAGGCAGGCACTAATCTGGAAATCACCATAATAACCCTCAA 5661
PV1             TGCCAAAGCGCTCGAAGATCAAGCAGGAACCAATCTTGAAATCACTATAATCACTCTAAA 5670
                 ** ***** *********** ***** ** ***** ******** ***** ** ** **

PV3             AAGAAATGAAAAGTTCAGAGATATCAGACAACACATACCCACTCAAATCACCGAGACGAA 5721
PV1             GAGAAATGAAAAGTTCAGAGACATTAGACCACATATACCTACTCAAATCACTGAGACAAA 5730
                 ******************** ** **** *** ***** *********** ***** **

PV3             TGATGGAGTTCTGATTGTGAACACTAGTAAGTACCCCAACATGTATGTTCCTGTCGGTGC 5781
PV1             TGATGGAGTCTTGATCGTGAACACTAGCAAGTACCCCAATATGTATGTTCCTGTCGGTGC 5790
                *********  **** *********** *********** ********************

PV3             TGTGACTGAGCAGGGATACCTAAATCTCGGTGGGCGCCAGACTGCTCGTATTCTAATGTA 5841
PV1             TGTGACTGAACAGGGATATCTAAATCTCGGTGGGCGCCAAACTGCTCGTACTCTAATGTA 5850
                ********* ******** ******************** ********** *********

PV3             CAACTTTCCAACCAGAGCTGGTCAGTGTGGTGGAGTCATCACATGCACTGGGAAAGTCAT 5901
PV1             CAACTTTCCAACCAGAGCAGGACAGTGTGGTGGAGTCATCACATGTACTGGGAAAGTCAT 5910
                ****************** ** *********************** **************

PV3             CGGGATGCACGTTGGTGGGAATGGTTCACATGGGTTTGCAGCGGCCCTGAAGCGGTCATA 5961
PV1             CGGGATGCATGTTGGTGGGAACGGTTCACACGGGTTTGCAGCGGCCCTGAAGCGATCATA 5970
                ********* *********** ******** *********************** *****
```

```
PV3             CTTCACTCAGAGCCAAGGTGAAATCCAGTGGATGAGACCATCAAAGGAGGCAGGGTATCC 6021
PV1             CTTCACTCAGAGTCAAGGTGAAATCCAGTGGATGAGACCTTCGAAGGAAGTGGGATATCC 6030
                ************ ************************** ** ***** *  ** *****

PV3             AATTATAAACGCCCCAACCAAGACCAAGCTCGAGCCCAGCGCTTTCCACTATGTGTTTGA 6081
PV1             AATCATAAATGCCCCGTCCAAAACCAAGCTTGAACCCAGTGCTTTCCACTATGTGTTTGA 6090
                *** ***** *****  **** ******** ** ***** ********************

PV3             AGGAGTAAAGGAACCAGCAGTCCTCACAAAGAATGATCCCAGACTCAAAACAGACTTTGA 6141
PV1             AGGGGTGAAGGAACCAGCAGTCCTCACTAAAAACGATCCCAGGCTTAAGACAGACTTTGA 6150
                *** ** ******************** ** ** ******** ** ** ***********

PV3             AGAAGCAATCTTCTCTAAGTATGTAGGGAACAAGATCACTGAGGTGGATGAGTACATGAA 6201
PV1             GGAGGCAATTTTCTCCAAGTACGTGGGTAACAAAATTACTGAAGTGGATGAGTACATGAA 6210
                 ** ***** ***** ***** ** ** ***** ** ***** *****************

PV3             AGAGGCAGTGGACCATTATGCTGGACAACTTATGTCGCTGGATATCAGCACAGAGCAAAT 6261
PV1             AGAGGCAGTAGACCACTATGCTGGCCAGCTCATGTCACTAGACATCAACACAGAACAAAT 6270
                ********* ***** ******** ** ** ***** ** ** **** ****** *****

PV3             GTGTCTAGAAGACGCCATGTATGGTACTGATGGTCTGGAGGCGCTAGATCTGTCTACCAG 6321
PV1             GTGCTTGGAGGATGCCATGTATGGCACTGATGGTCTAGAAGCACTTGATTTGTCCACCAG 6330
                ***  * ** ** *********** *********** ** ** ** *** **** *****

PV3             TGCCGGGTACCCCTACGTGGCAATGGGGAAGAAGAAGAGAGATATCCTAAACAAGCAAAC 6381
PV1             TGCTGGCTACCCTTATGTAGCAATGGGAAAGAAGAAGAGAGACATCTTGAACAAACAAAC 6390
                *** ** ***** ** ** ******** ************** *** * ***** *****

PV3             CAGAGACACCAAAGAAATGCAAAGACTTTTGGACGCTTACGGAATCAACCTACCATTAGT 6441
PV1             CAGAGACACTAAGGAAATGCAAAAACTGCTCGACACATATGGAATCAACCTCCCACTGGT 6450
                ********* ** ********** ***  * *** * ** *********** *** * **

PV3             GACATATGTCAAGGACGAGCTGAGGTCCAAAACAAAAGTGGAACAGGGAAAATCCAGACT 6501
PV1             GACTTATGTAAAGGATGAACTTAGATCCAAAACAAAGGTTGAGCAGGGGAAATCCAGATT 6510
                *** ***** ***** ** ** ** *********** ** ** ***** ********* *

PV3             GATTGAAGCTTCCAGTCTAAATGACTCAGTGGCCATGAGAATGGCATTTGGAAACCTTTA 6561
PV1             AATTGAAGCTTCTAGTTTGAATGACTCAGTGGCAATGAGAATGGCTTTTGGGAACCTATA 6570
                 *********** *** * ************** *********** ***** ***** **

PV3             TGCAGCATTCCACAGGAATCCAGGGGTCGTCACTGGTAGTGCAGTTGGATGCGATCCAGA 6621
PV1             TGCTGCTTTTCACAAAAACCCAGGAGTGATAACAGGTTCAGCAGTGGGGTGCGATCCAGA 6630
                *** ** ** ****  ** ***** **  * ** ***   ***** ** ***********

PV3             CCTATTCTGGAGCAAGATCCCAGTGTTGATGGAAGAAAAGCTATTTGCCTTTGATTACAC 6681
PV1             TTTGTTTTGGAGCAAAATTCCGGTATTGATGGAAGAGAAGCTGTTTGCTTTTGACTACAC 6690
                  * ** ******** ** ** ** *********** ***** ***** ***** *****

PV3             AGGATACGACGCATCACTTAGCCCAGCTTGGTTTGAGGCACTCAAGATGGTGTTAGAGAA 6741
PV1             AGGGTATGATGCATCTCTCAGCCCTGCTTGGTTCGAGGCACTAAAGATGGTGCTTGAGAA 6750
                *** ** ** ***** ** ***** ******** ******** ********* * *****

PV3             AATTGGTTTTGGAGATAGAGTGGATTACATAGACTACCTTAACCATTCACACCACTTGTA 6801
PV1             AATCGGATTCGGAGACAGAGTTGACTACATCGACTACCTAAACCACTCACACCACCTGTA 6810
                *** ** ** ***** ***** ** ***** ******** ***** ********* ****

PV3             CAAAAACAAGATATATTGTGTTAAGGGCGGCATGCCATCTGGCTGCTCCGGCACTTCAAT 6861
PV1             CAAGAATAAAACATACTGTGTCAAGGGCGGTATGCCATCTGGCTGCTCAGGCACTTCAAT 6870
                *** ** ** * *** ***** ******** ***************** ***********

PV3             TTTTAATTCAATGATTAACAATTTGATCATTAGGACGCTTTTACTGAAAACCTACAAGGG 6921
PV1             TTTTAACTCAATGATTAACAACTTGATTATCAGGACACTCTTACTGAAAACCTACAAGGG 6930
                ****** ************** ***** ** ***** ** ********************

PV3             CATAGATTTGGACCACTTAAAAATGATTGCCTATGGTGACGATGTAATAGCTTCCTATCC 6981
PV1             CATAGATTTAGACCACCTAAAAATGATTGCCTATGGTGATGATGTAATTGCTTCCTACCC 6990
                ********* ****** ********************** ******** ******** **

PV3             CCATGAGGTTGACGCTAGTCTCCTAGCCCAATCAGGAAAAGACTATGGACTAACCATGAC 7041
PV1             CCATGAAGTTGACGCTAGTCTCCTAGCCCAATCAGGAAAAGACTATGGACTAACTATGAC 7050
                ****** *********************************************** *****

PV3             TCCGGCAGATAAATCTGCCACTTTTGAGACAGTCACATGGGAGAATGTAACTTTCTTGAA 7101
PV1             TCCAGCTGACAAATCAGCTACATTTGAAACAGTCACATGGGAGAATGTAACATTCTTGAA 7110
                *** ** ** ***** ** ** ***** *********************** ********

PV3             AAGATTCTTCAGAGCAGATGAGAAATACCCCTTCCTCATACATCCAGTAATGCCAATGAA 7161
PV1             GAGATTCTTCAGGGCAGACGAGAAATACCCATTTCTTATTCATCCAGTAATGCCAATGAA 7170
                 *********** ***** *********** ** ** ** ********************

PV3             GGAAATTCATGAATCAATCAGATGGACAAAAGATCCTCGGAATACGCAGGACCATGTACG 7221
PV1             GGAAATTCATGAATCAATTAGATGGACTAAAGATCCTAGGAACACTCAGGATCACGTTCG 7230
                ****************** ******** ********* **** ** ***** ** ** **

PV3             CTCCTTGTGTCTATTGGCTTGGCACAACGGGGAAGAAGAATACAACAAATTTTTAGCTAA 7281
PV1             CTCTCTGTGCCTTTTAGCTTGGCACAATGGCGAAGAAGAATATAACAAATTCCTAGCTAA 7290
                ***  **** ** ** *********** ** *********** ********  *******
```

```
PV3             AATTAGGAGTGTGCCAATCGGAAGAGCTTTGTTGCTCCCAGAGTACTCAACATTGTACCG 7341
PV1             AATCAGGAGTGTGCCAATTGGAAGAGCTTTATTGCTCCCAGAGTACTCAACATTGTACCG 7350
                *** ************** *********** *****************************

PV3             CCGTTGGCTTGACTCATTTTAGTAACCCTACCTCAGTCGAATTGGATTGGGTCATACTGT 7401
PV1             CCGTTGGCTTGACTCATTTTAGTAACCCTACCTCAGTCGAATTGGATTGGGTCATACTGT 7410
                ************************************************************

PV3             TGTAGGGGTAAATTTTTCTTTAATTCGGAGG 7432
PV1             TGTAGGGGTAAATTTTTCTTTAATTCGGAG- 7440
                ******************************
```
