# Supplementary material for: Recombination in Enteroviruses Is a Biphasic Replicative Process Involving the Generation of Greater-than Genome Length ‘Imprecise’ Intermediates
Source: PLoS Pathog. 2014 Jun 12;10(6):e1004191. doi: 10.1371/journal.ppat.1004191 (PMC4055744; doi:10.1371/journal.ppat.1004191)
Supplement: Table S1 — Oligonucleotide primers used in the study. Degenerate sequences are indicated using standard IUPAC codes. (PDF) [file ppat.1004191.s007.pdf]

Table S1

Name	Sequence (5′ – 3′)
PV3-2995F	GCAAACATCTTCCAACCCGTCC
PV1/3-3280F	AACACRTMMGWGTCTGGTGC
GEN-4615R	RTADCCRTCRAAGTGWKMYGG
PV3-5191R	GGAGTCAACTGCTTGAAGCAGA
PV1-5200R	GGAGTCAACTGCTTGGAGCAAG
